# Distinct composition of different types of Abeta plaques in the pathogenesis of Alzheimer’s disease and the role of neutrophil-derived myeloperoxidase

**DOI:** 10.1186/s13041-025-01226-6

**Published:** 2025-06-22

**Authors:** Jianru Sun, Xiangqi Shao, Xue Wang, Xiang-sha Yin, Wenying Qiu, Xiaojing Qian, Fan Liu, Yongmei Chen, Chao Ma

**Affiliations:** 1 Department of Human Anatomy, Histology and Embryology, National Human Brain Bank for Development and Function, Institute of Basic Medical Sciences Chinese Academy of Medical Sciences, School of Basic Medicine Peking Union Medical College, No. 5 Dongdansantiao, Dongcheng District, Beijing, 100005 P. R. China; 2 State Key Laboratory of Common Mechanism Research for Major Diseases, Institute of Basic Medical Sciences, Chinese Academy of Medical Sciences and Peking Union Medical College, Beijing, P. R. China; 3 Neuroscience Center, , Institute of Basic Medical Sciences Chinese Academy of Medical Sciences, School of Basic Medicine Peking Union Medical College, Beijing, P. R. China; 4 Chinese Institute for Brain Research, Beijing, 102206 P. R. China

**Keywords:** Amyloid plaques, Neutrophil granulocytes, MPO, ECog score, ABC score, Neuroimmune

## Abstract

Alzheimer’s disease (AD) is an age-related neurodegenerative disorder. Different types of Aβ plaques are likely to play distinct roles in the brains of patients with AD. In this study, through the combination of pathological techniques and analysis of the human brain database, we discovered that focal Aβ plaques (FAPs), rather than diffuse Aβ plaques (DAPs), are significantly correlated with AD-related neuropathological changes and cognitive impairment. By using laser capture microdissection in conjunction with microproteomics, the protein components of different Aβ plaques were characterized. Bioinformatic analysis indicated that FAP-enriched proteins are associated mainly with immune-related pathways, such as neutrophil extracellular trap formation. We further confirmed that myeloperoxidase (MPO) is significantly upregulated in the AD brain and colocalizes with FAPs but not with DAPs. Immunohistochemical staining demonstrated that neutrophils expressing MPO accumulated in the capillary lumen and brain parenchyma. The number of neutrophils significantly increases in the cortex and hippocampus of AD donors. Our study revealed a potential role for neutrophil-derived MPO in FAPs, providing insights into the pathogenesis mechanisms and potential therapeutic targets of AD.

**Supplementary Information:**

The online version contains supplementary material available at 10.1186/s13041-025-01226-6.

## Introduction

Alzheimer’s disease (AD) is an age-related neurodegenerative disorder. One of the typical pathologic features of AD is senile plaques formed by the extracellular deposition of β-amyloid (Aβ) [1]. Aβ is generated by proteolytic cleavage of amyloid precursor protein (APP) by β- and γ-secretases [2]. Aβ peptides of different lengths can be converted into oligomeric and protofibrillar forms and eventually form Aβ plaques [3, 4]. As the main component of plaques, the Aβ peptide can induce neuronal death through the activation of glial cells [5]. However, studies have shown that the severity of AD symptoms does not scale linearly with the quantity of plaques in the brain, and Aβ plaques are also found in healthy elderly individuals without apparent cognitive impairment [6]. To date, various AD therapeutics for the clearance of Aβ have not achieved the anticipated effects; therefore, systematic studies of the composition of Aβ plaques are essential.

According to the postmortem neuropathology of the human brain, Aβ plaques have different morphologies and are mainly divided into diffuse and focal (or dense) types [7–9]. Although electron microscopy analysis of postmortem human brains revealed that all forms of Aβ plaques are associated with neuropathology, significant distinctions exist between diffuse Aβ plaques (DAPs) and focal Aβ plaques (FAPs). Research has indicated that FAPs might induce neuronal damage and microglial activation, phenomena closely linked to pathological alterations in AD [10, 11]. We have also reported that FAPs in the hippocampus are significantly associated with AD pathology, cognitive impairment and neuroinflammation [12]. Therefore, further studies of the differences between FAPs and DAPs in the human brain are necessary. Our research group used quantitative proteomics to investigate the differences in Aβ plaques in the AD human brain, the aging human brain and the APP/PS1 mouse brain and reported that synaptic structural proteins and complement proteins are upregulated in the Aβ plaques of the AD human brain [13]. However, the proteins identified in this study are relatively limited, and there is a paucity of omics studies targeting different types of plaques.

Owing to disruption of the blood‒brain barrier in AD pathology, cells, including peripheral macrophages, neutrophils, T cells, NK cells and B cells, can infiltrate the human and mouse brain [14–17]. Studies have revealed that in AD patients, neutrophils can obstruct capillaries and lead to a decrease in blood flow but are seldom detected in the brain parenchyma [18]. In AD mouse models, neutrophils adhere to cerebral capillaries, which reduces cortical blood flow and impairs memory function [19, 20]. Research has shown that neutrophils play a role in AD; however, the precise mechanisms by which neutrophils contribute to AD pathogenesis and their specific impact on Aβ pathology remain to be elucidated.

In this study, through analysis of various regions of the human cerebral cortex and hippocampus, we discovered that FAPs rather than DAPs are significantly correlated with AD-related neuropathological changes and cognitive impairment. We further characterized the composition of different Aβ plaque forms via laser capture microdissection in conjunction with microproteomics. Striking differences were observed between the DAPs and FAPs. We found that FAP-enriched proteins are associated mainly with immune-related pathways, such as neutrophil extracellular trap formation. Immunohistochemical staining demonstrated that neutrophils expressing myeloperoxidase (MPO) accumulated in the capillary lumen and brain parenchyma. The number of neutrophils in the cortex and hippocampus of AD donors significantly increases with increasing severity of AD. We further confirmed that the MPO protein is significantly upregulated in the AD brain and colocalizes with FAPs but not with DAPs. Our study revealed a role for MPO expressed by neutrophils in FAPs in the AD brain, providing insights into the pathogenesis mechanisms and potential therapeutic targets of AD.

## Methods

### Human brain sample sources

A total of 106 fixed human brain samples in 10% formalin, which were obtained from the National Human Brain Bank for Development and Function at the Chinese Academy of Medical Sciences/Peking Union Medical College (CAMS/PUMC), were used in this research. Detailed information, including demographic variables, ABC scores, ECog scores and other information on all donors, is listed in Supplementary Table 1. The research protocol was approved by the Institutional Review Board of the Institute of Basic Medical Sciences of the Chinese Academy of Medical Sciences, China (Approval Numbers: 009–2014, 031–2017, and 2022125). All antemortem written informed consent forms were received from both the potential donor and his/her next of kin to guarantee that the donation was completely voluntary and that ethically approved use of the brain tissues in future scientific research was permitted. The brain tissues were collected from 2012 to 2023 following published standard human brain banking procedures [21].

### Neuropathological evaluation

According to the National Institute on Aging and Alzheimer’s Association (NIA-AA) guidelines [22] the brain tissues all underwent identical neuropathological analysis via the ABC score, which was conducted by professionals at the Brain Bank. The ABC score was used to assess the presence of Aβ deposits (A score), neurofibrillary tangles (B score), and neuritic plaques (C score). The general ABC score was divided into four distinct categories on the basis of the ADNC: None (N), Low (L), Intermediate (I), and High (H). The ADNC diagnosis of “N/L” suggests that it is unlikely for the donor to have AD. In this case, we can consider the donors to be normal elderly individuals. On the other hand, the ADNC diagnosis of the “I/H” group indicated a high probability of AD in the donor. Therefore, the “I/H” group provides substantial evidence for dementia [22].

The brain regions, including the middle frontal gyrus (MFG), superior and middle temporal gyri (SMTG), inferior parietal lobule (IPL), occipital cortex (OC), hippocampus (HP), basal ganglia, cerebellar cortex, dentate, and midbrain, were sampled from postmortem human brains. These tissues were fixed in 10% formalin and subsequently embedded in paraffin. For the pathological staining of A and B, the paraffin-embedded brain tissues were sectioned to a thickness of 5 μm for A and B scoring, whereas a thickness of 10 μm was used for C scoring. Immunohistochemical staining was performed to detect Aβ deposits via an anti-Aβ antibody (mouse monoclonal antibody, 1:200, DAKO #M0872), whereas neurofibrillary tangles (NFTs) were detected via an anti-p-Tau (Ser202, Thr205) antibody (mouse monoclonal antibody, 1:800, Thermo #MN1020) [23]. The membranes were incubated with primary antibodies overnight at 4 °C, followed by processing with a mouse two-step detection kit (mouse enhanced polymer detection system) (ZSGB-BIO #PV-9002) for 60 min. Visualization of the staining results was achieved via a DAB chromogenic kit (ZSGB-BIO #ZLI-9019). C-scores were determined through a modified Bielschowsky staining method targeting neuritic processes in senile plaques [24].

### Cognitive function assessment

Clinical cognitive status was determined via the ECog Insider Questionnaire, a 39-question assessment aimed at evaluating the antemortem daily cognitive function of brain donors. The ECog questionnaire consists of six subitems: memory, language, visuospatial functions, planning, organization, and divided attention [25, 26]. An average ranging from 39 to 156 was calculated for both the global ECog score and the separate score for each ECog domain. Face-to-face or phone interviews with the immediate kin of the brain donor were conducted for all the subjects recruited from the CAMS/PUMC brain tissue bank. The related items were marked ‘‘unknown’’ if the informant could not recall or respond. If more than half of the items were marked ‘‘unknown’’, the average score was not calculated. Ecog scores from 98 human brain donors were collected and used for subsequent analysis.

### Laser capture microdissection of plaque samples

Cerebral cortex tissues from 3 donors with “L/I” ABC scores were utilized for DAP capture, whereas cerebral cortex tissues from 3 age-matched donors with “I/H” ABC scores were used for FAP capture. To capture Aβ plaques, small blocks of cerebral cortex tissues were embedded in OCT embedding medium (Tissue-Tek^®^ O.C.T., Sakura #4583), frozen on dry ice, and stored at -80 °C. Brain sections (35 μm thick) were cut using a Leica CM 3050 cryostat (Leica Microsystems Inc., Bannockburn, IL) and mounted on PET membrane slides (Molecular Machines and Industries GmbH, Eching, Germany). The frozen brain sections were thawed at room temperature and stained with the anti-Aβ antibody 6E10 (mouse monoclonal antibody, 1:200, BioLegend #803015) according to the manufacturer’s instructions. Laser capture microdissection was performed on the same day via the MMI CellCut system (Molecular Machines and Industries GmbH, Eching, Germany) with the following settings: cut velocity, 136 μm/sec; laser focus, 160 μm; and laser power, 100%. Regions with diameters of 90–110 μm centered on an Aβ plaque were collected. Approximately 1000 Aβ plaques were procured from each brain sample.

### Peptide enzymatic digestion and mass spectrometry

Captured samples of the same group were transferred to Eppendorf tubes, ensuring that the samples were at the bottom of the Eppendorf tubes. The sample was treated with 5 µl of BT lysis buffer, 10 µl of 50 mM ammonium bicarbonate (ABC) solution, and 1% phenylmethanesulfonyl fluoride (PMSF) to ensure full coverage. Noncontact ultrasonication was subsequently conducted on the sample at 4 °C for 10 min. Following ultrasonication, a brief centrifugation was performed to settle the solution at the bottom of the tube. The sample was then subjected to heating at 90 °C and 1500 rpm for 15 min, with centrifugation every 5 min to prevent evaporation. After heating, a brief centrifugation was carried out, and the sample was cooled to room temperature. Next, 3.7 µl of 50 mM dithiothreitol (DTT) was added, followed by incubation at 55 °C for 30 min. Subsequently, 3.5 µl of 100 mM iodoacetamide (IAA) solution was added, and the reaction was conducted in the dark at room temperature for 30 min. Then, 20 µl of 50 mM ammonium bicarbonate (ABC) solution was added to reduce the final concentration of BT to less than 0.5%. Finally, trypsin was added for digestion at an enzyme-to-protein mass ratio of 1:10, with digestion carried out at 37 °C under shaking at 1500 rpm. The peptides were quantified via a Nanodrop spectrophotometer after enzymatic digestion. Each component was separated via a nanoElute liquid chromatography system from Bruker. Mobile phase A consisted of a 0.1% formic acid solution in water, while mobile phase B contained 0.1% formic acid in acetonitrile. The gradient elution conditions were as follows: 0–45 min, 2–22% B; 45–50 min, 22–37% B; 50–55 min, 37–80% B; and 55–60 min, 80% B. After chromatographic separation by the ultrahigh-performance liquid chromatography system, the peptides were injected into a TOF Pro mass spectrometer manufactured by Bruker for analysis. The mass spectrometry parameters were set as follows: the capillary voltage was maintained at 1400 V, with primary and secondary scan ranges ranging from 100 to 1700 m/z and ion mobility windows (1/K0) ranging from 0.7 to 1.4 Vs/cm^2^.

### Proteome analysis

To process the original data-independent acquisition (DIA) data, Spectronaut Pulsar software was used with fixed carbamidomethyl © modification, variable oxidation (M), and acetyl (N-term) modifications, allowing for a maximum of 2 missed cleavage sites. Differentially expressed proteins needed to meet the criteria of *P* < 0.05 and a fold change ≥ 1.5 or a fold change ≤ 1/1.5, where *P* < 0.05 and a fold change ≥ 1.5 indicated significant upregulation, whereas *P* < 0.05 and a fold change ≤ 1/1.5 indicated significant downregulation. Differential protein analysis of BPs, CCs, and MFs was performed via the GO database. The KEGG database was used to investigate the primary pathways related to the differentially expressed proteins. Furthermore, the PPI analysis was based on the STRING database, leading to the development of a differential protein interaction network.

### Immunohistochemistry and immunofluorescence staining

Formalin-fixed frontal cortex tissues were embedded in OCT embedding medium (Tissue-Tek^®^ O.C.T., Sakura #4583), frozen on dry ice, and stored at -80 °C. Brain Sect. (14 μm thick) were cut using a Leica CM 3050 cryostat (Leica Microsystems Inc., Bannockburn, IL). Double immunofluorescence staining was used to label Aβ and HLA class II histocompatibility antigen DR (HLA-DR), complement C3 (C3) or MPO. Immunohistochemical staining was used to label MPO, S100 calcium binding protein A8 (S100A8) and CD66b. For the colabeling of Aβ and neutrophil markers (S100A8 or CD66b), Aβ immunohistochemistry was performed first, followed by immunofluorescence staining for neutrophil markers. The frozen tissue sections were incubated separately overnight at 4 °C with primary antibodies (rabbit anti-HLA-DR, 1:200, Abcam #ab92511; rabbit anti-C3, 1:1000, Abcam #ab200999; rabbit anti-MPO, 1:500, Abcam #ab208670; rabbit anti-S100A8, 1:200, Proteintech Group #15792-I-AP; rabbit anti-CD66b, 1:10000, Abcam #ab300122; mouse anti-Aβ, 1:200, DAKO #M0872) and then incubated with the proper secondary antibodies (Alexa Fluor 594-conjugated goat anti-rabbit, 1:400, Abcam #ab150080; Alexa Fluor 488-conjugated goat anti-mouse, 1:400, Abcam #ab150113; or mouse two-step detection kit, ZSGB-BIO #PV-9002). A DAB chromogenic kit (ZSGB-BIO #ZLI-9019) was added after incubation with a mouse two-step detection kit. The slides were then washed in PBS and cover-slipped with Mounting Medium with DAPI (Abcam #ab104139). Images of the sections were obtained via a microscopic imaging system (Olympus BX61 and FluoView software).

Using ImageScope software, we conducted immunohistochemical analysis of Aβ in ten 10X-microscopic fields of the MFG, SMTG, IPL, OC, and HP of the human brain. The sum of MFG, SMTG, IPL and OC represents the quantity of Aβ plaques in the cerebral cortex. We divided Aβ plaques into two types: DAPs and FAPs. Plaques that looked like loose structures with irregular and ill-defined margins were defined as DAPs. Plaques that had clear-cut outlines and generally had compact cores or strong sepia granular textures were defined as FAPs. Then, the percentage of each plaque type can be calculated.

### Immunoblotting

Brain samples were harvested with RIPA lysis buffer (CWBIO #CW2333S) supplemented with 100x protease inhibitor cocktail (CWBIO #CW2200S) and 100x phosphatase inhibitor cocktail (CoWin #CW2383S). The methods used to prepare the brain extract samples are explained below. The samples were spun down at 15,000 × g for 15 min at 4 °C on a tabletop centrifuge, and then the supernatant was collected for protein quantification via a Pierce BCA protein assay kit. The samples were prepared via 5x SDS‒PAGE loading buffer (CWBIO #CW0027S) according to the manufacturer’s instructions and then run on 10% SDS‒PAGE gels with 1x Tris‒glycine SDS buffer (CWBIO #CW0045S). Western blot transfer was performed on ice via the addition of 1x Tris‒glycine transfer buffer (CWBIO #CW0044S) onto a polyvinylidene fluoride membrane, which was subsequently blocked for 1 h at room temperature with 5% bovine serum albumin. The membranes were incubated with primary antibodies diluted in blocking buffer overnight at 4 °C (MPO, 1:1000; Abcam #ab208670). Washes were performed with TBS buffer containing 0.01% Tween-20 (TBST). The blots were incubated with secondary antibodies diluted in TBS-T buffer (goat anti-rabbit IgG, HRP conjugated, 1:3000; CWBIO #CW0103S) for 1 h at room temperature. Washes were performed again in TBST before imaging using an LICOR Odyssey CLx imager. The bands were quantified via ImageJ, and the signals were normalized to the actin levels.

### Statistical analysis

The values are expressed as the means ± SEMs. Statistical analyses were conducted via SPSS software (version 17.0) and GraphPad software. Student’s t test was used to analyze the statistical significance of differences between 2 groups, and one-way analysis of variance (ANOVA) followed by Scheffe’s post hoc test was used to analyze the statistical significance of differences among 3 groups. The relationships between different types of data and the ABC score were analyzed via Spearman correlation analysis. *p* < 0.05 was considered significant. The relationships between different types of data and the ABC score were analyzed via Spearman correlation analysis. *p* < 0.05 was considered significant. The relationships between different types of data and the ABC score were analyzed via Pearson correlation analysis. *p* < 0.05 was considered significant.

## Results

### Associations between brain Aβ plaque form and demographic variables

**Fig. 1:**
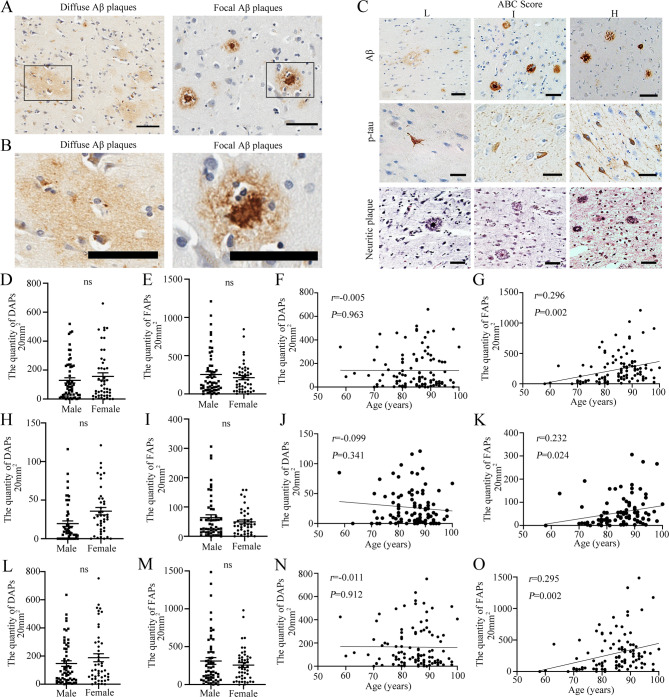
Correlations between demographic variables and Aβ plaque forms. **A** Histochemical staining morphology of DAPs and FAPs from Aβ plaque-positive human brain tissue. **B** Enlarged image of DAP and FAP in human brain tissue. **C** Neuropathologic assessment of the ABC score for the Aβ plaque-positive human brain. In **A-C**, scale bar = 50 μm. **D-E** Quantification of DAPs (**E**), FAPs (**F**) in the cerebral cortex of different genders. male (*n* = 59), female (*n* = 47), male group versus female group, not significant, by Student’s t test. **F**,** J** and **N** The correlation between the quantity of DAPs in the cerebral cortex (**F**), hippocampus (**J**) or whole brain (**N**) and age. *p* > 0.05, no significant correlation, by Pearson correlation. **G** The correlation between the quantity of FAPs in the cerebral cortex and age. *p* = 0.002, *r* = 0.296, significant positive correlation, by Pearson correlation. **H-I** Quantification of DAPs (**H**), FAPs (**I**) in the hippocampus of different genders. male (*n* = 53), female (*n* = 42), male group versus female group, not significant, by Student’s t test. **K** The correlation between the quantity of FAPs in the hippocampus and age. *p* = 0.024, *r* = 0.232, significant positive correlation, by Pearson correlation. **L-M** Quantification of DAPs (**L**), FAPs (**M**) in the whole brain across different genders. male (*n* = 59), female (*n* = 47), male group versus female group, not significant, by Student’s t test. **O** The correlation between the quantity of FAPs in the whole brain and age. *p* = 0.002, *r* = 0.295, significant positive correlation, by Pearson correlation. In **F**, **G**, **N** and **O**, 106 samples were included. In **J** and **K**, 95 samples were included

Immunohistochemistry revealed that human brain Aβ plaques exist in different forms in Aβ plaque-positive human brain samples (Fig. 1A-B). As outlined in our previous article [12] the human brain contains two predominant subtypes of Aβ plaques—DAPs and FAPs—which are categorized based on their distinct morphologies. Immunostaining revealed that the DAPs were loose structures with irregular, ill-defined margins, whereas the FAPs had clear outlines and generally had a core. FAPs are extracellular proteinaceous deposits primarily consisting of Aβ peptides, predominantly in the form of amyloid filaments, whereas DAPs contain a majority of nonaggregated proteins without amyloid filaments. It is important to better understand the differences between FAPs and DAPs.

The associations of demographic variables with the quantity of total Aβ plaques in the human brain cortex (Fig. S 1 A-C), HP (Fig. S 1D-F) and whole brain (Fig. S 1G-I) were investigated separately. No statistically significant difference in the quantity of total Aβ plaques was found based on sex (Student’s t test, *p* > 0.05) (Fig. S 1 A, D and G). There was also no significant difference in the quantity of total Aβ plaques between the different age groups (one-way ANOVA, *p* > 0.05) (Fig. S 1B, E and H). Pearson correlation analysis revealed a weak positive correlation between the quantity of total Aβ plaques and age in both the cortex and the whole brain (Fig. S1C and I). However, no significant correlation was found between the quantity of total Aβ plaques and age in the HP (Fig. S 1F). The associations between demographic variables and different forms of Aβ plaques in the human brain cortex (Fig. 1D-G), HP (Fig. 1H-K) and whole brain (Fig. 1L-O) were also examined individually. There were no significant differences in the quantity of DAPs or FAPs based on sex (Student’s t test, *p* > 0.05) (Fig. 1D, E, H, I, L and M). Pearson correlation analysis revealed no statistically significant associations between the quantity of DAPs and age (Fig. 1F, J and N). The correlation between the quantity of FAPs and age was weakly positive (Fig. 1G, K and O). These results suggest that as age increases, the number of FAPs in the human brain tends to increase.

### Correlation between brain Aβ plaque formation and AD-related neuropathologic changes

**Fig. 2:**
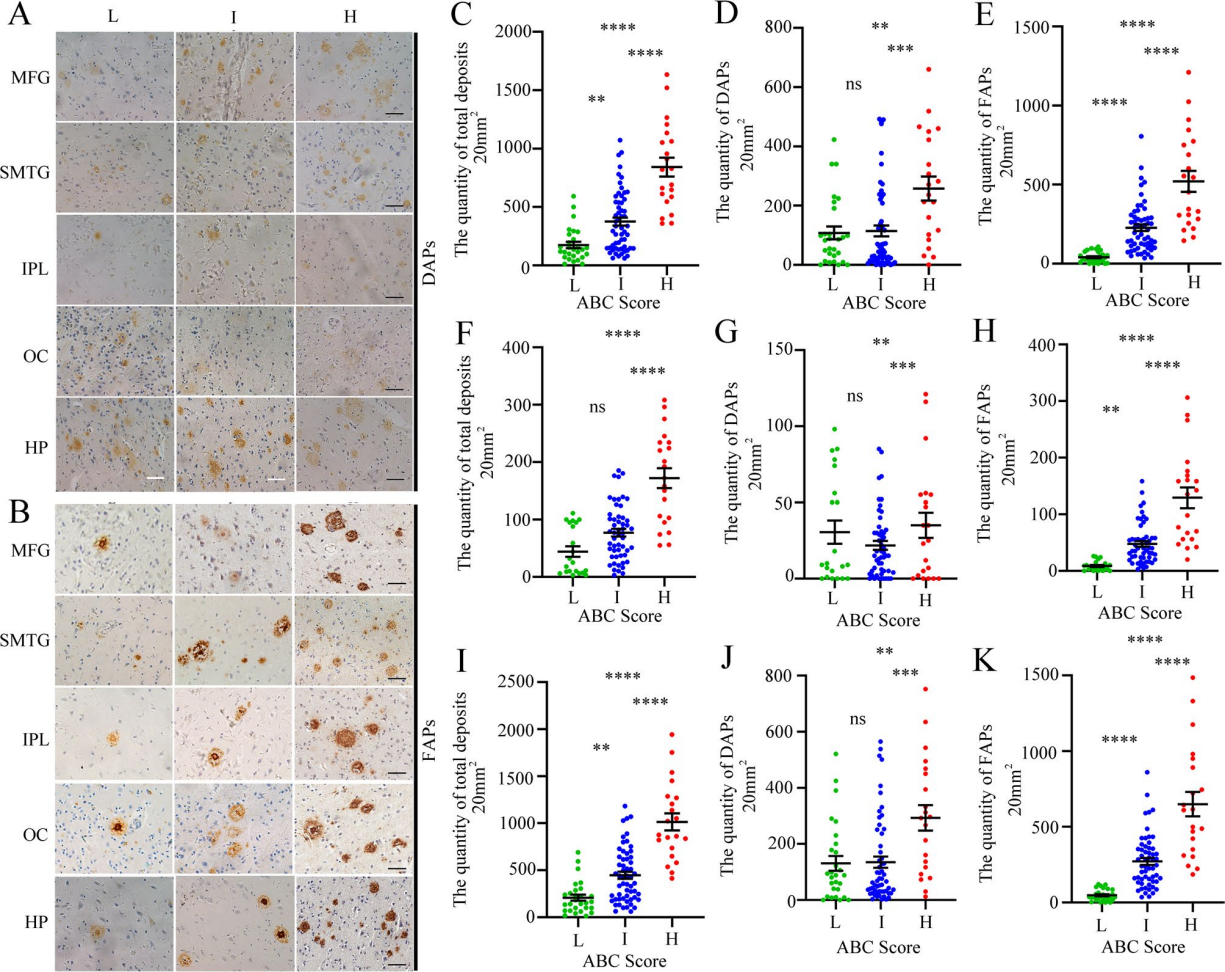
The quantity of FAPs increases with the degree of AD neuropathologic change. **A** Distribution of DAPs in the human brain across different ABC score groups. **B** Distribution of FAPs in the human brain across different ABC score groups. In **A-B**, scale bar = 50 μm. MFG: middle frontal gyrus, SMTG: superior and middle temporal gyri, IPL: inferior parietal lobule, OC: occipital cortex, HP: hippocampus. **C**-**E** The quantity of total Aβ plaques (**C**), DAPs (**D**) or FAPs (**E**) in the cerebral cortex across different ABC score groups. **F-H** The quantity of total Aβ plaques (**F**), DAPs (**G**) or FAPs (**H**) in the hippocampus across different ABC score groups. **I-K** The quantity of total Aβ plaques in the whole brain (**I**), DAPs (**J**) or FAPs (**K**) across different ABC score groups. In **C**-**E** and **I**-**K**, “L” group (*n* = 28), “I” group (*n* = 57) and “H” group (*n* = 21). In **F**-**H**, “L” group (*n* = 21), “I” group (*n* = 53) and “H” group (*n* = 21), **p < 0.01, ***p < 0.001, ****p < 0.0001, by one-way ANOVA followed by Scheffe’s post hoc test

Immunohistochemistry experiments revealed that more FAPs were observed in the intermediate (“I” ABC score) or high-level (“H” ABC score) AD neuropathologic brain samples across various regions, including the MFG, SMTG, IPL, OC and HP, whereas the DAPs were not observed (Fig. 2A-B). The neuropathological assessment of AD in brain samples was conducted according to ABC scores, which included Aβ deposits, neurofibrillary tangles (p-Tau) and neuritic plaques. Figure 1C shows immunohistochemical images of Aβ plaques, p-Tau and neuritic plaques in different ABC score (“L”, “I” and “H”) groups. We conducted a statistical analysis of the variations in Aβ plaque quantity within the cerebral cortex (Fig. 2C-E), HP (Fig. 2F-H), and whole brain (Fig. 2I-K) across different ABC score groups. In the cerebral cortex, one-way ANOVA revealed that the quantity of total Aβ plaques in the “I” and “H” ABC score groups was significantly greater than that in the “L” ABC score group (Fig. 2C). Further analysis revealed that the quantity of FAPs, not DAPs, significantly increased with increasing ABC score in the cerebral cortex. Specifically, the quantity of FAPs in the “I” and “H” ABC score groups was significantly greater than that in the “L” ABC score group (Fig. 2E), whereas the number of DAPs did not significantly differ between the “L” and “I” ABC score groups but increased only slightly in the “H” ABC score group (Fig. 2D). In the HP, one-way ANOVA revealed that the quantity of total Aβ plaques in the “H” ABC score group was significantly greater than that in the “L” and “I” ABC score groups (Fig. 2F). The quantity of FAPs in the HP also significantly increased with increasing ABC score (Fig. 2H), whereas the number of DAPs only slightly increased in the “H” group (Fig. 2G). In the whole brain, the trend in the number of Aβ plaques across the groups was consistent with that observed in the cortex (Fig. 2I-K).

**Fig. 3:**
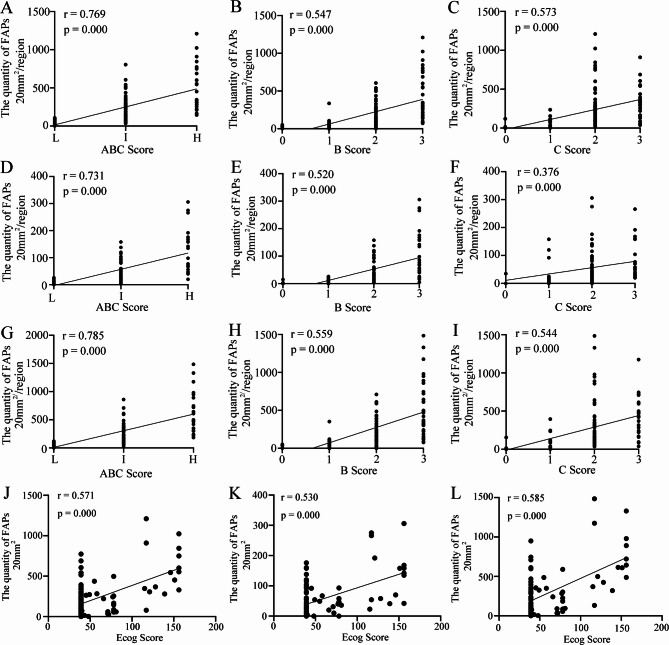
Correlations between FAPs and ABC scores or ECog scores in the human brain. **A** The correlation between the quantity of FAPs in the cerebral cortex and the ABC score. *p* = 0.000, *r* = 0.769, significant positive correlation, by Spearman correlation. **B** The correlation between the quantity of FAPs in the cerebral cortex and the B score. *p* = 0.000, *r* = 0.547, significant positive correlation, by Spearman correlation. **C** The correlation between the quantity of FAPs in the cerebral cortex and the C score. *p* = 0.000, *r* = 0.573, significant positive correlation, by Spearman correlation. **D** The correlation between the quantity of FAPs in the hippocampus and the ABC score. *p* = 0.000, *r* = 0.731, significant positive correlation, by Spearman correlation. **E** The correlation between the quantity of FAPs in the hippocampus and the B score. *p* = 0.000, *r* = 0.520, significant positive correlation, by Spearman correlation. **F** The correlation between the quantity of FAPs in the hippocampus and the C score. *p* = 0.000, *r* = 0.376, significant positive correlation, by Spearman correlation. **G** The correlation between the quantity of FAPs in the whole brain and the ABC score. *p* = 0.000, *r* = 0.785, significant positive correlation, by Spearman correlation. **H** The correlation between the quantity of FAPs in the whole brain and the B score. *p* = 0.000, *r* = 0.559, significant positive correlation, by Spearman correlation. **I** The correlation between the quantity of FAPs in the whole brain and the C score. *p* = 0.000, *r* = 0.544, significant positive correlation, by Spearman correlation. In **A-C** and **G-I**, L group = 28 samples, I group = 57 samples and H group = 21 samples. In **D-F**, L group = 21 samples, I group = 53 samples and H group = 21 samples. **J** The correlation between the quantity of FAPs in cerebral cortex and ECog score. *p* = 0.000, *r* = 0.571, significant positive correlation, by Spearman correlation. **K** The correlation between the quantity of FAPs in hippocampus and ECog score. *p* = 0.000, *r* = 0.530, significant positive correlation, by Spearman correlation. **L** The correlation between the quantity of FAPs in the whole brain and ECog score. *p* = 0.585, *r* = 0.000, significant positive correlation, by Spearman correlation. In **J** and **L**, 98 samples were included. In **K**, 87 samples were included

Spearman rank correlation analysis was used to evaluate the correlation between the quantity of different Aβ plaque forms in the human brain and AD neuropathological scores. In the cerebral cortex, there was a moderate positive correlation between the quantity of FAPs and the ABC score (*r* = 0.769, *p* = 0.000) (Fig. 3A), B score (*r* = 0.547, *p* = 0.000) (Fig. 3B) and C score (*r* = 0.573, *p* = 0.000) (Fig. 3C). However, the correlation between the quantity of DAPs and the ABC score was only very weakly positive (*r* = 0.245, *p* = 0.011) (Fig. S 2 A), and there was no significant correlation between the quantity of DAPs and the B score (*r* = 0.152, *p* = 0.120) (Fig. S 2B) or C score (*r* = 0.149, *p* = 0.127) (Fig. S 2 C). In the HP, the quantity of FAPs exhibited a moderate positive correlation with the ABC score (*r* = 0.731, *p* = 0.000) (Fig. 3D) and B score (*r* = 0.520, *p* = 0.000) (Fig. 3E) and a weak positive correlation with the C score (*r* = 0.376, *p* = 0.000) (Fig. 3F). In contrast, there was no significant correlation between DAP quantity and the ABC score (*r* = 0.051, *p* = 0.624) (Fig. S 2D), B score (*r* = -0.058, *p* = 0.577) (Fig. S 2E), or C score (*r* = 0.101, *p* = 0.331) (Fig. S 2 F). In the whole brain, a moderate positive correlation was also observed between the quantity of FAPs and the ABC score (*r* = 0.785, *p* = 0.000) (Fig. 3G), B score (*r* = 0.559, *p* = 0.000) (Fig. 3H), and C score (*r* = 0.544, *p* = 0.000) (Fig. 3I). However, the correlation between the quantity of DAPs and the ABC score was only very weakly positive (*r* = 0.265, *p* = 0.006) (Fig. S 2G), with no significant correlation found between the quantity of DAPs and the B score (*r* = 0.141, *p* = 0.148) (Fig. S 2 H) or C score (*r* = 0.169, *p* = 0.084) (Fig. S 2I). The above results further indicate that FAPs, rather than DAPs, participate in neuropathological changes in AD.

We investigated the distribution of different Aβ plaques across cortical functional areas, including the MFG, OC, IPL, and SMTG, and revealed a sequential increase in the quantity of FAPs rather than DAPs with increasing ABC score (Fig. S 3 A-D). The correlations between the quantity of distinct plaque types in each brain functional area and the ABC score, B score, and C score were consistent with the aforementioned findings (Table 1).

**Table 1:** The correlations between the quantity or percentage of different Aβ plaque forms in brain areas and the AD pathological score

	Region	The correlation between the quantity of different types of Aβ plaques and AD pathological score				The correlation between the percentage of different types of Aβ plaques and AD pathological score		
		ABC	B	C		ABC	B	C
Diffuse plaques	MFG	0.153^ns^	0.097^ns^	0.088^ns^		-0.016^ns^	-0.013^ns^	-0.021^ns^
	SMTG	0.247^*^	0.171^ns^	0.182^ns^		0.029^ns^	-0.031^ns^	0.030^ns^
	IPL	0.306^**^	0.201^*^	0.153^ns^		0.028^ns^	-0.053^ns^	-0.032^ns^
	OC	0.139^ns^	0.091^ns^	0.057^ns^		-0.027^ns^	-0.025^ns^	-0.031^ns^
	Cortex	0.245^*^	0.152^ns^	0.149^ns^		-0.246^*^	-0.258^**^	-0.186^ns^
	HP	0.164^ns^	0.009^ns^	0.085^ns^		-0.095^ns^	-0.209^*^	0.039^ns^
Focal plaques	MFG	0.551^****^	0.392^****^	0.351^***^		0.312^**^	0.288^**^	0.268^**^
	SMTG	0.574^****^	0.432^****^	0.444^****^		0.297^**^	0.315^***^	0.261^**^
	IPL	0.551^****^	0.431^****^	0.418^****^		0.293^**^	0.222^*^	0.340^***^
	OC	0.637^****^	0.513^****^	0.453^****^		0.400^****^	0.338^***^	0.264^**^
	Cortex	0.769^****^	0.547^****^	0.573^****^		0.309^**^	0.310^**^	0.272^**^
	HP	0.735^****^	0.582^****^	0.383^****^		0.419^****^	0.365^***^	0.183^ns^

The results are displayed as correlation coefficients (*p* values) in each cell. MFG = middle frontal gyrus, SMTG = superior and middle temporal gyri, IPL = inferior parietal lobule, OC = occipital cortex, HP = hippocampus. *p < 0.05, **p < 0.01, ***p < 0.001, ****p < 0.0001, by Spearman correlation

### Associations between brain Aβ plaque formation and cognitive dysfunction

Spearman correlation analysis was used to confirm the relationships between different Aβ plaque forms and cognitive dysfunction in the human brain. In the cerebral cortex, the quantity of FAPs, rather than DAPs, demonstrated a moderately positive correlation with the everyday cognitive (ECog) score (*r* = 0.571, *p* = 0.000) (Fig. 3J). The quantity of FAPs in the HP was also positively correlated with the ECog score (*r* = 0.530, *p* = 0.000) (Fig. 3K). The quantity of FAPs in the whole brain also exhibited a significant positive correlation with the ECog score (*r* = 0.585, *p* = 0.000) (Fig. 3L). The correlation between the quantity of distinct plaque types in each brain functional area and the Ecog score was consistent with the aforementioned findings (Table 2). To further evaluate the correlation between different Aβ plaque forms and cognitive dysfunction, a multiple linear regression model was established with the quantity of FAPs and the quantity of DAPs in the whole brain as independent variables and the Ecog score as the dependent variable. The results demonstrated a significant effect of FAPs on the ECog score (β = 0.070, *p* < 0.001), whereas no significant effect was detected for the DAPs (β = 0.019, *p* = 0.292) (Table 3). Considering the influence of age on cognition, consistent results were obtained when age was included as a confounding factor (Table 3).

**Table 2:** The correlation between the quantity of different Aβ plaque forms in brain areas and the global Ecog score

	Region	Global Ecog score
Diffuse plaques	MFG	0.210^*^
	SMTG	0.248^*^
	IPL	0.269^**^
	OC	-0.095^ns^
	Cortex	0.266^**^
	HP	0.049^ns^
Focal plaques	MFG	0.422^****^
	SMTG	0.610^****^
	IPL	0.463^****^
	OC	0.343^***^
	Cortex	0.571^****^
	HP	0.537^****^

The results are displayed as correlation coefficients (*p* values) in each cell. MFG = middle frontal gyrus, SMTG = superior and middle temporal gyri, IPL = inferior parietal lobule, OC = occipital cortex, HP = hippocampus. *p < 0.05, **p < 0.01, ***p < 0.001, ****p < 0.0001, by Spearman correlation

**Table 3:** Linear regression for the quantity of different Aβ plaque forms and the Ecog score

Variables	Multivariate model 1*				Multivariate model 2*		
	β	β0	*P*		β	β0	*P*
The quantity of diffuse plaques	0.019	36.213	0.292		0.019	43.524	0.313
The quantity of focal plaques	0.07	36.213	< 0.001		0.071	43.524	< 0.001

The dependent variable is ECog score. The independent variable is FAPs and DAPs. Age (confounding factor) was included in multivariate model 2. β: Slope coefficients. β0: Intercept coefficient. *P* = *P* value

### Quantitative proteomics reveals the distinct composition of different Aβ plaque forms in the human brain

DAPs from the human cortex of the “L/I” group and FAPs from the human cortex of the “I/H” ABC score group were collected via laser capture microdissection after immunohistochemical staining of frozen sections. After pretreatment of the ultramicro sample and quantification of the peptide segment level, the timsTOF Pro2 mass spectrometry platform was used for DIA mode acquisition to obtain the original data. The original data are then subjected to direct DIA spectrum matching and quantitative information extraction. A total of 4508 quantified proteins were detected in all the samples, including 4471 in DAPs and 4487 in FAPs. The quality assessment of the proteomics data ensures the reliability of the subsequent bioinformatic analyses (Fig. S4A-E).

**Fig. 4:**
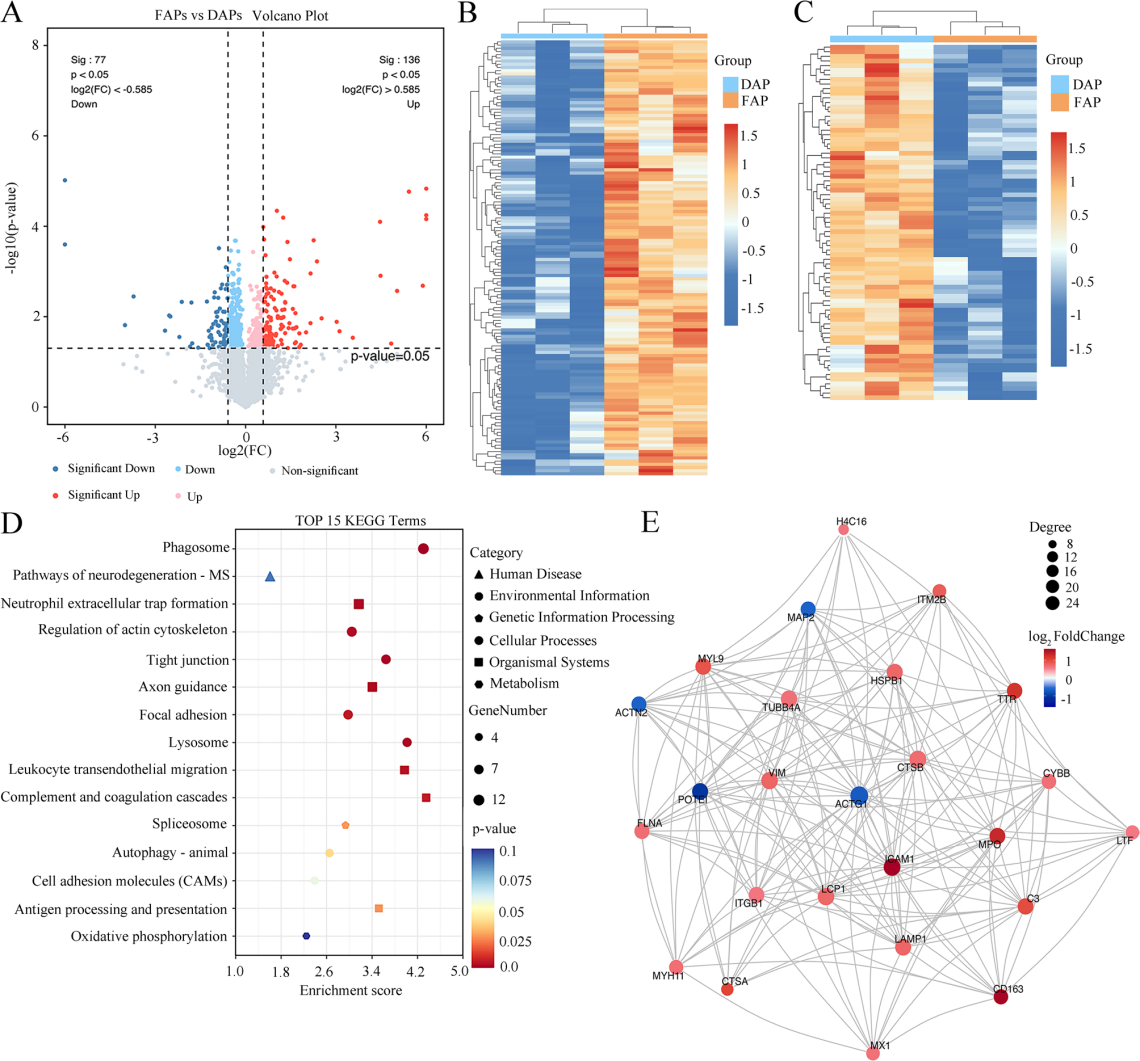
Differences in protein profiles between human brain FAPs and DAPs. **A** Volcano plot showing the differences in the expression of human brain FAPs and DAPs. The light blue dots represent the 77 proteins whose expression was significantly downregulated in FAPs compared with that in DAPs (log2(FC) < -0.585, *p* value < 0.05). The light red dots represent the 136 proteins whose expression was significantly upregulated in FAPs compared with that in DAPs (log2(FC) > 0.585, *p* value < 0.05). log2(FC) = log2(fold change). **B** Hierarchical clustering heatmap of all upregulated protein expression levels in human brain FAPs versus DAPs, represented by colored boxes after Z score conversion. **C** Hierarchical clustering heatmap of all downregulated protein expression levels in human brain FAPs versus DAPs, represented by colored boxes after Z score conversion. **D** Top 15 enriched KEGG pathways of upregulated proteins in FAPs. **E** The PPI network of the differentially expressed proteins between FAPs and DAPs. The panel displays the Top25 connectivity protein interaction network diagram, where circles represent differential proteins. Red indicates upregulation, and blue indicates downregulation, with the circle size reflecting the level of connectivity; larger circles denote greater connectivity

Striking differences were observed between different forms of Aβ plaques. Compared with those in the DAPs, 136 proteins in the FAPs were significantly increased (fold change ≥ 1.5, *p* value < 0.05), and 77 proteins in the FAPs were significantly decreased (fold change ≤ 1/1.5, *p* value < 0.05) (Fig. 4A). Figure 4B shows 136 upregulated proteins in human brain FAPs compared with those in DAPs. Figure 4C shows that 77 proteins were downregulated in human brain FAPs compared with those in DAPs. Gene Ontology Biological Process (GOBP), Gene Ontology Cellular Component (GOCC) and Gene Ontology Molecular Function (GOMF) enrichment analyses were performed on all differentially expressed proteins identified between FAPs and DAPs. Fig. S5A illustrates the top 45 GO pathways of differentially expressed proteins in FAPs compared to DAPs, involving various biological processes. GOBP, GOCC, and GOMF enrichment analyses were performed on the upregulated proteins in FAPs. A bubble chart displaying the top 45 GO terms is shown in Fig. S5B. The top 15 GOBP terms included processes such as complement activation, positive regulation of tumor necrosis factor production, defense response, antibacterial humoral response, substrate adhesion-dependent cell spreading and extracellular matrix disassembly. The four most enriched GOCC terms were extracellular exosome, extracellular region, extracellular space and cell surface. Kyoto Encyclopedia of Genes and Genomes (KEGG) enrichment analysis further revealed that the upregulated proteins of FAPs were mainly involved in pathways such as phagosome, neutrophil extracellular trap formation, complement and lysosome pathways (Fig. 4D). These results indicate that the upregulated proteins of FAPs mainly participate in immune-related pathways, playing significant roles in key biological processes such as phagocytosis, neutrophil extracellular trap formation, the complement cascade, and cell adhesion. Apart from immune-related proteins, the upregulated proteins in FAPs encompass multiple functional categories. These include Erlin-2 (ERLIN2) and tripeptidyl-peptidase 1 (TPP1), which are associated with lipid metabolism; Desmin (DES), Scinderin (SCIN), and Stathmin-4 (STMN4), which contribute to cytoskeleton reorganization; apoptosis regulator BAX (BAX), neuroglobin (NGB), and heat shock protein beta-1 (HSPB1), which play roles in apoptosis regulation; and Netrin-1 (NTN1) and Roundabout homolog 2 (ROBO2), which are involved in axon guidance. Additionally, Integral membrane protein 2B (ITM2B) and ITM2C, which negatively regulate the biosynthesis of amyloid precursor protein, are also upregulated in FAPs. Fig. S6A and Fig. S6C illustrate the proteins exclusively expressed in FAPs and their involvement in KEGG pathways.

Further GO enrichment analysis of the downregulated proteins in FAPs revealed that these proteins were significantly enriched in several GOBP pathways, including protein transport, endocytosis, the mitochondrial oxidative respiratory chain, proteinase activity regulation, and actin cytoskeleton organization, which are associated with diverse cellular biological processes (Fig. S5C). The KEGG enrichment analysis results further confirmed that these downregulated proteins were involved in multiple key signaling pathways (Fig. S5D). Fig. S6B and Fig. S6D respectively presented the proteins expressed only in DAPs and the KEGG pathways they participated in.

The PPI network of differentially expressed proteins in FAPs and DAPs was constructed using the STRING database (http://string-db.org). This network comprises 25 nodes. By employing the Network Analyzer plugin in Cytoscape software, the network was analyzed based on topological features, including node degree and betweenness centrality. The results revealed that the top 10 key proteins with the highest node degree were: cathepsin B (CTSB), intercellular adhesion molecule 1 (ICAM1), vimentin (VIM), plastin-2 (LCP1), tubulin beta-4 A chain (TUBB4A), HSPB1, integrin beta-1 (ITGB1), C3, and MPO (Fig. 4E).

### Quantitative proteomics revealed that immune-related proteins are enriched in faps

**Fig. 5:**
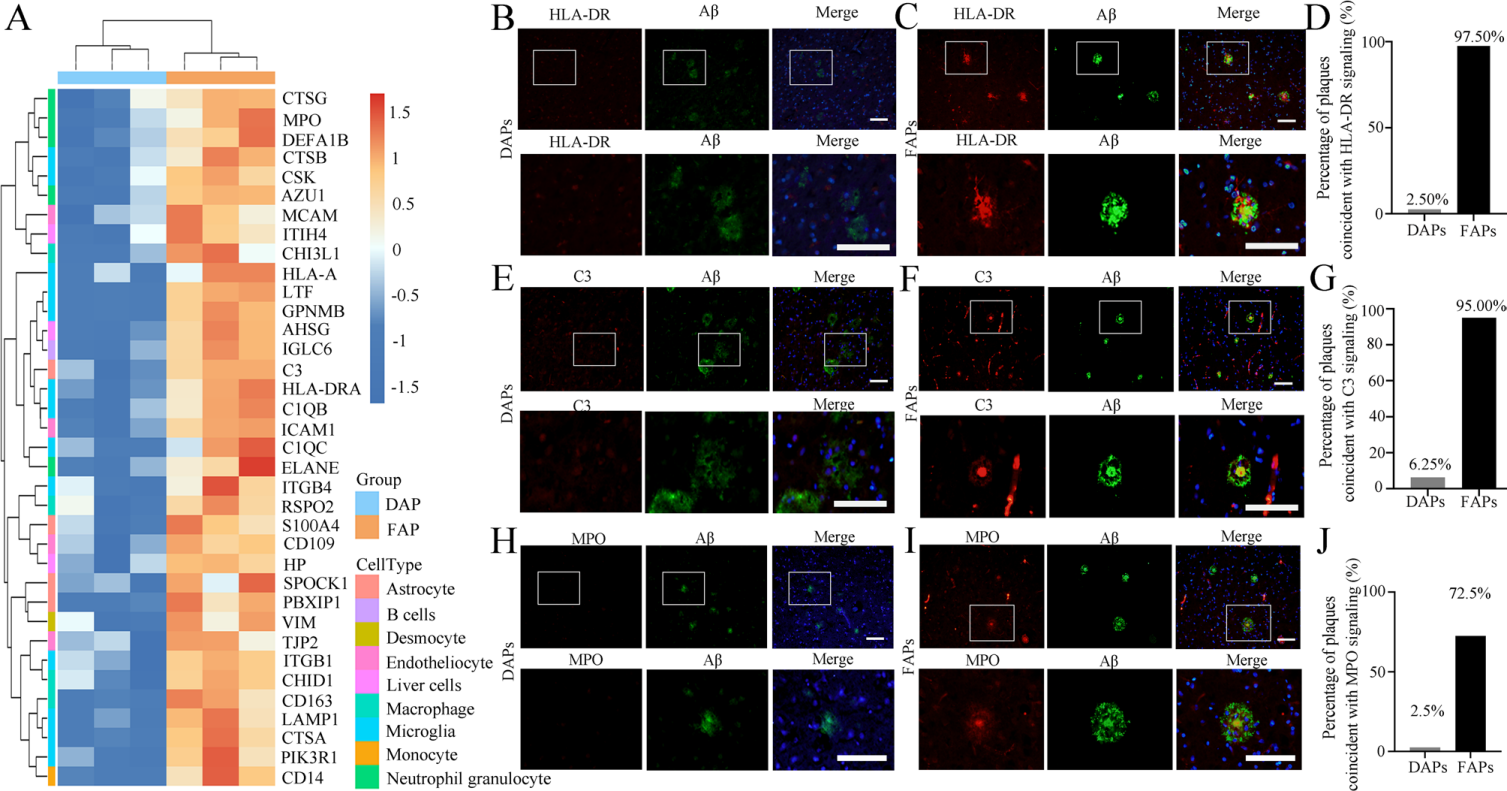
Validation of significantly upregulated immune-related proteins in human brain FAPs versus DAPs. **A** Hierarchical clustering heatmap of 36 upregulated immune-related protein expression levels in human brain FAPs, represented by colored boxes after Z score conversion. **B-C** Representative images of double immunofluorescence staining for HLA-DR and DAPs (**B**) or FAPs (**C**) in human brain cortex. **D.** Percentages of different types of plaques co-localized with HLA-DR signaling. *n* = 80 for each plaque type from 6 human brain samples covering the “L/I/H” group. **E-F** Representative images of double immunofluorescence staining for C3 and DAP (**E**) or FAP (**F**) in human brain cortex. **G** Percentages of different types of plaques co-localized with C3 signaling. *n* = 80 for each plaque type from 6 human brain samples covering the “L/I/H” group. **H-I** Representative images of double immunostaining for MPO and DAPs (**H**) or FAPs (**I**) in human brain cortex. **J** Percentages of different types of plaques co-localized with MPO signaling. *n* = 80 for each plaque type from 6 human brain samples covering the “L/I/H” group. In **B**,** C**,** E**,** F**,** H** and **I**, enlarged images of the regions outlined by solid-line boxes are shown below. Scale bar = 100 μm

We normalized the expression values of 36 upregulated immune-related proteins in FAPs and generated an expression heatmap (Fig. 5A). Through cell type-specific analysis, we identified that among these 36 upregulated immune-related proteins, 13 were predominantly expressed by microglia, 5 by neutrophils, and 4 by astrocytes. Specifically, microglia-associated proteins included HLA-DR, complement C1q (C1Q), and lysosome-associated membrane glycoprotein 1 (LAMP1), etc.; neutrophil-associated proteins included MPO, azurocidin (AZU1), neutrophil elastase (ELANE), cathepsin G (CTSG) and neutrophil defensin 1 (DEFA1B); and astrocyte-associated proteins include C3, S100 calcium-binding protein A4 (S100A4). SPOCK1, PBXIP1. These findings provide critical insights into the cellular origins and functional properties of immune-related proteins in FAPs. We independently verified the enrichment results of HLA-DR, C3 and MPO in FAPs.

HLA-DR serves as a key antigen-presenting molecule on the surface of microglia, and its expression is closely associated with the activation state of cellular immunity. Notably, during the pathological progression of AD, HLA-DR is significantly upregulated [27–29] indicating that the immune response mediated by HLA-DR in microglia may contribute to the pathogenesis of AD. To validate our proteomic data, we performed dual immunofluorescence staining for Aβ plaques and HLA-DR. The results demonstrated that FAPs exhibited significant co-localization with HLA-DR-positive signals, whereas no comparable co-localization was observed in DAPs (Fig. 5B-C). We randomly selected 80 FAPs and 80 DAPs from human brain samples and performed a statistical analysis of their HLA-DR-positive signals. The results revealed that 78 (97.5%) of the FAPs exhibited HLA-DR-positive signals, whereas only 2 (2.5%) of the DAPs displayed such signals (Fig. 5D). C3 is expressed mainly by astrocytes, plays a pivotal role in the complement cascade. and is increased in AD patients [30]. It colocalizes with neuritic plaques and appears to facilitate the clearance of Aβ by microglia in the brain [31, 32]. Our staining results demonstrated that C3 can be significantly colocalized with FAPs, whereas DAPs do not show such positivity. Furthermore, the intensity of the C3 signal is directly proportional to the density of FAPs (Fig. 5E-F). We randomly selected 80 FAPs and 80 DAPs and reported that 76 (95.0%) FAPs presented positive C3 signals, whereas only 5 (6.25%) DAPs presented positive C3 signals (Fig. 5G).

MPO is a peroxidase that is stored in the granules of neutrophils and is released into the extracellular space during degranulation [33, 34]. It is a marker of neutrophil activation and fights against the invasion of pathogens as an antimicrobial protein in natural immunity [34]. Our immunofluorescence staining of MPO and Aβ plaques revealed that FAPs, especially those in the cores of the plaques, presented strong MPO-positive signals, whereas DAPs did not (Fig. 5H-I). The colocalization of Aβ plaques with MPO was statistically analyzed based on the MPO staining results. Eighty plaques of each type were randomly selected, and the statistical results revealed that 58 (72.5%) of the FAPs exhibited MPO-positive signals, whereas only 2 (2.5%) of the DAPs displayed such signals (Fig. 5J).

### The number of neutrophils in the human brain significantly increases with increasing severity of AD

**Fig. 6:**
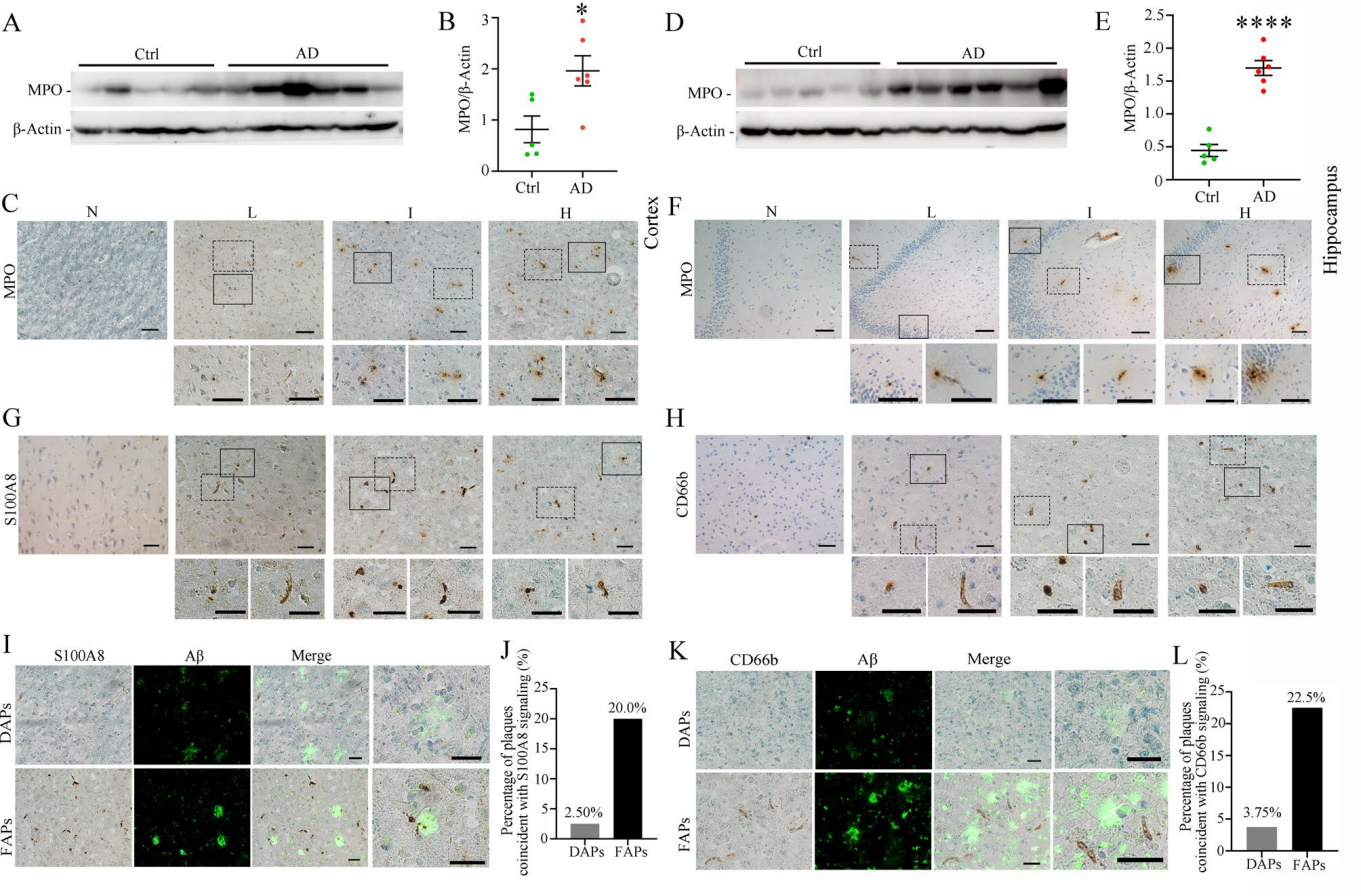
Neutrophil numbers are significantly increased and associated with FAPs in the AD human brain. **A** Western blot of MPO in the human brain cortex. **B** The relative expression levels of MPO with β-Actin as the internal reference in the human brain cortex in different groups. **C** Distribution of MPO-positive signals in the human brain cortex across ABC score groups. **D** Western blot of MPO in the human hippocampus. **E** The relative expression levels of MPO with β-Actin as the internal reference in the human hippocampus in different groups. In **B** and **E**, data are shown as the mean ± SEM. *p < 0.05, ****p < 0.0001, by Student’s t test. Ctrl group: ABC score “N” and “L” (*n* = 5). AD group: ABC score “I” and “H” (*n* = 6). **F** Distribution of MPO-positive signals in the human hippocampus across ABC score groups. In **C** and **F**, scale bar = 100 μm. **G** Distribution of S100A8-positive signals in the human brain cortex across ABC score groups. **H** Distribution of CD66b-positive signals in the human brain cortex across ABC score groups. In **C**,** F**,** G** and **H**, the solid-line boxes and dashed-line boxes respectively highlight neutrophils in the brain parenchyma and those within the vascular lumen. The corresponding magnified images are shown below. Groups: “N” (*n* = 6), “L” (*n* = 6), “I” (*n* = 6) and “H” (*n* = 6). **I** Representative images of double immunostaining for S100A8 and DAPs or FAPs in the human brain. **K** Representative images of double immunostaining for CD66b and DAPs or FAPs in the human brain. **J** and **I** Percentages of different types of plaques associated with S100A8 (**J**) and CD66b (**I**) signaling. *n* = 80 for each plaque type from 6 human brain samples covering the “L/I/H” group. In **G-H**,** I** and **K**, scale bar = 50 μm

Given that the FAP-enriched proteins MPO, AZU1, ELANE, CTSG and DEFA1B are all traditionally believed to be expressed mainly by neutrophils, we focused on exploring the relationship between neutrophils and AD pathology. Previous studies have verified that MPO is almost exclusively expressed by neutrophils in the human brain and can be a specific marker of activated neutrophils. Western blotting revealed that MPO in the human cerebral cortex was significantly greater in the “I/H” group than in the “N/L” group (Fig. 6A-B). Immunohistochemical staining revealed a substantial quantity of MPO-positive neutrophils in the human brain cortex, with their numbers significantly correlating with the severity of AD pathology (Fig. 6C). We also concluded that MPO-positive neutrophils were present in two distinct forms: those that had infiltrated into the brain parenchyma (parenchymal infiltration) and those located within the brain vasculature (intravascular retention). The protein expression level of MPO in the hippocampus of AD donors was also markedly elevated, and the number of MPO-positive neutrophils was increased (Fig. 6D-F). To further investigate the relationship between neutrophils and AD pathology, we performed immunohistochemical staining using two traditional neutrophil markers, S100A8 and CD66b. The results demonstrate that as the severity of AD pathology increases, the number of S100A8- and CD66b-positive neutrophils also increases correspondingly. Additionally, these neutrophils exhibit two distinct forms: parenchymal infiltration and intravascular retention (Fig. 6G-H). The colocalization of these two markers with Aβ plaques further indicates that FAPs, rather than DAPs, are closely associated with neutrophils (Fig. 6I-L). Our findings reveal a significant increase in neutrophil quantity within the brain tissue of AD donors, presenting in two distinct forms: parenchymal infiltration and intravascular retention. Additionally, neutrophil-derived MPO was markedly upregulated in the brains of AD donors and demonstrated significant co-localization with FAPs.

## Discussion

Different types of Aβ plaques exist in the brains of humans with AD. An in-depth exploration of the roles that different plaques play in AD is vital. We previously studied the correlations between different Aβ plaque forms and AD pathology and cognitive dysfunction. However, these studies were limited to plaques in the hippocampal region. The deposition sequence of amyloid proteins first occurs in the cortex, and a more comprehensive study of Aβ plaques in different brain regions is needed. In this study, we systematically analyzed the spatial distribution of different subtypes of Aβ plaques across functional regions of the cerebral cortex and HP on the basis of pathology-based data from 106 Chinese AD donors. The results revealed that in the cerebral cortex, HP and entire brain, the quantity of FAPs significantly increased with increasing ABC score, whereas the number of DAPs did not significantly differ between the “L” and “I” ABC score groups but increased only slightly in group “H”. We further investigated the associations of distinct types of Aβ plaques in the cortex, HP and entire brain with AD neuropathological scores and donor cognitive scores. We concluded that in both the cerebral cortex and HP, FAPs, rather than DAPs, participated in AD neuropathological changes (ABC score, B score, C score) and cognitive dysfunction (ECog score). These findings suggest that in the human brain, FAPs, rather than DAPs, may contribute to neuropathological changes in AD. Furthermore, FAPs in the human brain may serve as potential indicators of cognitive function.

Previous proteomic studies on AD have focused mainly on different brain tissues, and the differentially expressed proteins are involved in AD-related pathways, such as the amyloid cascade, inflammation, WNT signaling, lipid metabolism, iron homeostasis, and membrane transport pathways [35–38]. Our research group previously employed quantitative proteomics to characterize the distinct features of Aβ plaques in the brains of AD patients, aged individuals, and APP/PS1 mice, revealing the specific upregulation of synaptic structural proteins and complement proteins within Aβ plaques in AD human brains [13]. However, the scope of identified proteins is relatively restricted, and omics studies specifically focusing on different types of Aβ plaques are still lacking. Therefore, we employed laser microdissection in combination with microproteomics to investigate the compositional differences of FAPs and DAPs and their surrounding tissues in human brain samples, as well as to identify Aβ plaque-associated proteins implicated in the AD mechanism. We identified over 4,000 proteins associated with Aβ plaques and their surrounding microenvironment. Compared with DAPs, FAPs exhibited 136 upregulated proteins and 77 downregulated proteins, indicating significant differences in protein composition between FAPs and DAPs. KEGG and GO enrichment analysis revealed that the upregulated proteins in FAPs were predominantly enriched in immune-related pathways, such as phagosome pathways, neutrophil extracellular trap formation, and complement pathways. These findings suggest that immune responses may play a critical role in FAP-related pathologies. Further analysis demonstrated that a group of immune-related proteins were enriched in FAPs, including 13 proteins primarily expressed by microglia, 5 proteins primarily expressed by neutrophils, and 4 proteins primarily expressed by astrocytes.

Among the differential proteins, some have been recognized as risk factors for AD, and some have been proven to play important roles in the pathogenesis of AD. Taking the complement system as an example, previous studies have demonstrated both beneficial and harmful roles of complement in Aβ-related neuropathology. Microglial C1Q can upregulate its expression in response to pathological AD proteins, enhancing microglial phagocytosis and exacerbating synaptic loss in amyloid mouse models [39, 40]. C3, which is predominantly synthesized by astrocytes, can also be induced to increase in response to Aβ peptides, colocalizing with neuritic plaques and facilitating Aβ clearance by microglia [31, 41]. Our mass spectrometry data further confirmed the potential significant involvement of complement in Aβ plaque pathology, and complement exhibited exclusive colocalization with FAPs. Importantly, our immunofluorescence staining of human brain tissue revealed a direct correlation between the intensity of the C3 signal and FAP density. Further investigations into the mechanisms by which C1Q and C3 participate in FAP pathology are warranted.

We focused particularly on the upregulation of neutrophil-related proteins in FAPs. Five neutrophil-associated proteins enriched in FAPs are MPO, AZU1, NE, CTSG, and DEFA1B. These proteins act as molecular markers of neutrophil activation and are stored in azurophilic granules. Upon cell activation, they are released into the extracellular space through degranulation and contribute to antibacterial defense, inflammation regulation, and tissue remodeling. Among these neutrophil-related proteins, MPO is a crucial antibacterial protein for neutrophil function. Previous studies have demonstrated significantly elevated plasma MPO levels [42, 43] and MPO-immunoreactive cells or extracellular MPO in brain regions [18, 44] among AD patients, suggesting MPO may serve as a biomarker and potential therapeutic target for AD. Our proteomics data demonstrate that MPO expression in FAPs is significantly higher than in DAPs (fold change = 2.49, *p* < 0.01). PPI network analysis of differentially expressed proteins further indicates its central role in the molecular network associated with FAPs. Additionally, we confirm that MPO expression is upregulated in the cerebral cortex and hippocampus of AD donors and significantly co-localizes with FAPs. Our study demonstrates the potential role of MPO in Aβ pathology, especially in FAPs. This discovery offers new insights into the heterogeneity of Aβ plaques in AD and the mechanisms of neutrophil-mediated neuroinflammation.

By performing immunohistochemical staining for MPO, CD66b and S100A8, we discovered that there were numerous neutrophils in the cerebral cortex and hippocampus of AD donors and that the quantity of neutrophils was positively correlated with the severity of AD pathology. These findings indicate that the blood‒brain barrier is impaired in the AD state. Our study also demonstrated that there are two forms of neutrophils in the human brain: those that have infiltrated into the brain parenchyma and those located within the capillary lumen. Previous research has shown that neutrophils block capillaries in AD patients, leading to reduced blood flow, but researchers seldom found neutrophils in the brain parenchyma [18]. Our findings supplement and update previous conclusions and further indicate the role of neutrophils in AD. We also found that neutrophils colocalized with FAPs rather than with DAPs. The colocalization of neutrophils and FAPs implies that neutrophils might play an important role in the formation and progression of Aβ pathology.

Our study has certain limitations. Future research should focus on further in-depth investigations to determine whether neutrophils and their derived MPO directly influence Aβ pathology, as well as to elucidate the precise mechanisms involved. To achieve this, gene knockout mice and overexpression models can be constructed to systematically validate the regulatory role of MPO in Aβ pathology and uncover its underlying mechanisms. Moreover, depletion experiments targeting neutrophils in AD model mice could provide valuable insights into the role of neutrophils in AD.

In this study, we discovered that FAPs, rather than DAPs, are significantly correlated with AD-related neuropathological changes and cognitive impairment in the human cerebral cortex and hippocampus. We characterized the composition of DAPs and FAPs via laser capture microdissection and microproteomics, revealing striking differences between DAPs and FAPs. FAP-enriched proteins are associated mainly with immune-related pathways, such as neutrophil extracellular trap formation. We further confirmed that MPO is upregulated in AD brains and colocalizes with FAPs but not with DAPs. The number of neutrophils increases significantly in the cortex and hippocampus of AD donors, and neutrophils accumulate in the capillary lumen and brain parenchyma. Our findings suggest a potential role for neutrophil-derived MPO in FAPs, providing insights into the pathogenesis mechanisms and potential therapeutic targets of AD.

## Electronic supplementary material

Below is the link to the electronic supplementary material.

Supplementary Material 1

## Data Availability

All data are available in the main text or the supplementary material of this article.
