# Supplementary material for: Distinct composition of different types of Abeta plaques in the pathogenesis of Alzheimer’s disease and the role of neutrophil-derived myeloperoxidase
Source: Mol Brain. 2025 Jun 22;18:53. doi: 10.1186/s13041-025-01226-6 (PMC12182686; doi:10.1186/s13041-025-01226-6)
Supplement: Supplementary file 1 — Supplementary Material 1 [file 13041_2025_1226_MOESM1_ESM.docx]

**Supplementary Figures**


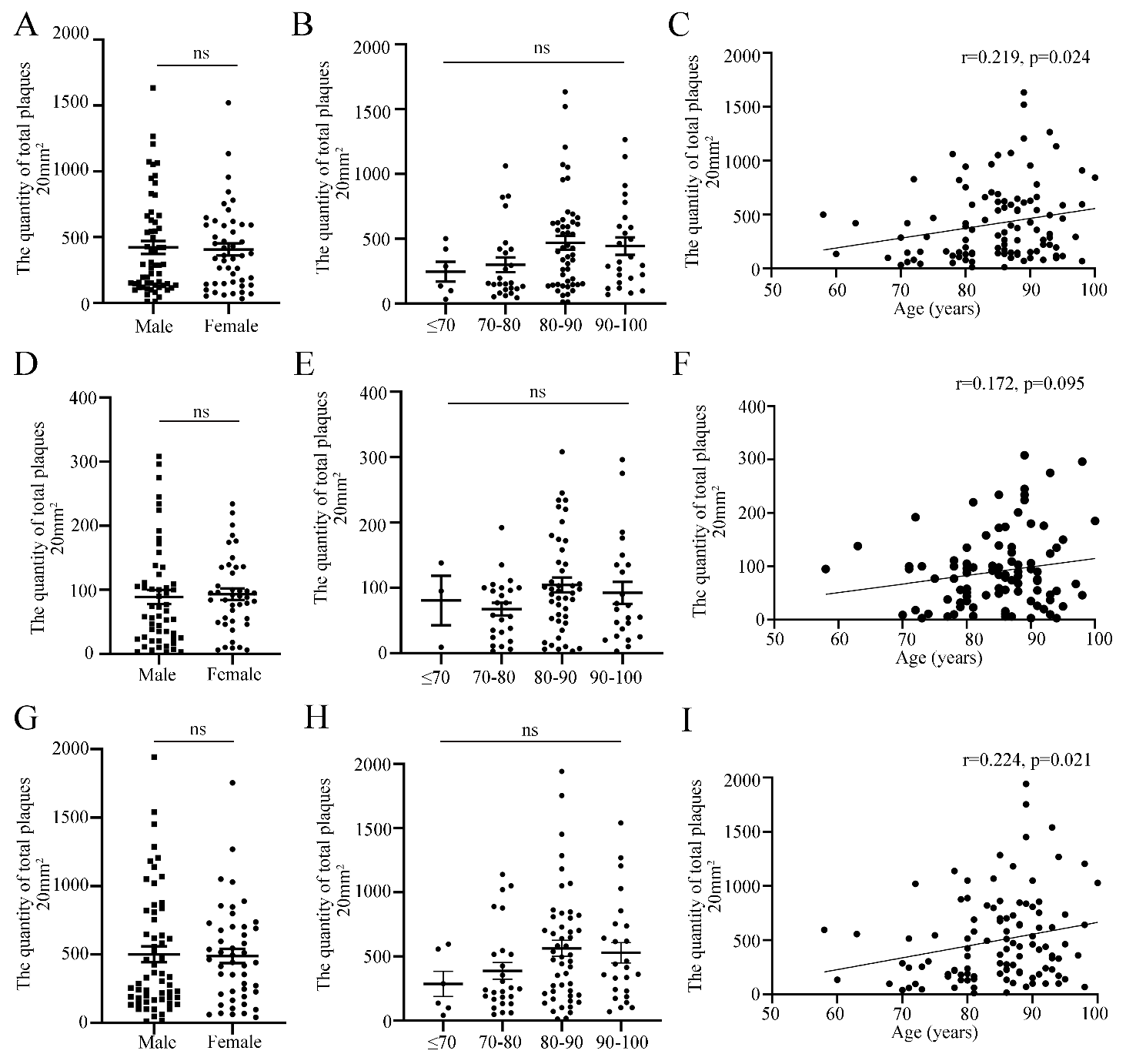


**Figure S1. Correlation between total A****β plaques and demographic variables.** **A** Quantification of total Aβ plaques in cerebral cortex of different genders. male (n = 59), female (n = 47), male group versus female group, not significant, by Student's t test. **B** Quantification of total Aβ plaques in cerebral cortex of different age groups. ≤70 years (n = 6), 70-80 years (n = 26), 80-90 years (n = 49), and 90-100 years (n = 25). not significant, by one-way ANOVA followed by Scheffe’s post-hoc test. **C** The correlation between the quantity of total Aβ plaques in the whole brain and age. *p* = 0.024, r = 0.219, significant positive correlation, by Pearson correlation. **D** Quantification of total Aβ plaques in hippocampus of different genders. male (n = 53), female (n = 42), male group versus female group, not significant, by Student's t test. **E** Quantification of total Aβ plaques in hippocampus of different age groups. ≤70 years (n = 3), 70-80 years (n = 24), 80-90 years (n = 44), and 90-100 years (n = 23). not significant, by one-way ANOVA followed by Scheffe’s post-hoc test. **F** The correlation between the quantity of total Aβ plaques in hippocampus and age. *p* > 0.05, no significant correlation, by Pearson correlation. **G** Quantification of total Aβ plaques in the whole brain of different genders. male (n = 59), female (n = 47), male group versus female group, male group versus female group, not significant, by Student's t test. **H** Quantification of total Aβ plaques in the whole brain of different age groups. ≤70 years (n = 6), 70-80 years (n = 26), 80-90 years (n = 49), and 90-100 years (n = 25). not significant, by one-way ANOVA followed by Scheffe’s post-hoc test. **I** The correlation between the quantity of total Aβ plaques in the whole brain and age. *p* = 0.021, r = 0.224, significant positive correlation, by Pearson correlation. In **C** and **I**, 106 samples were included. In **F**, 95 samples were included.


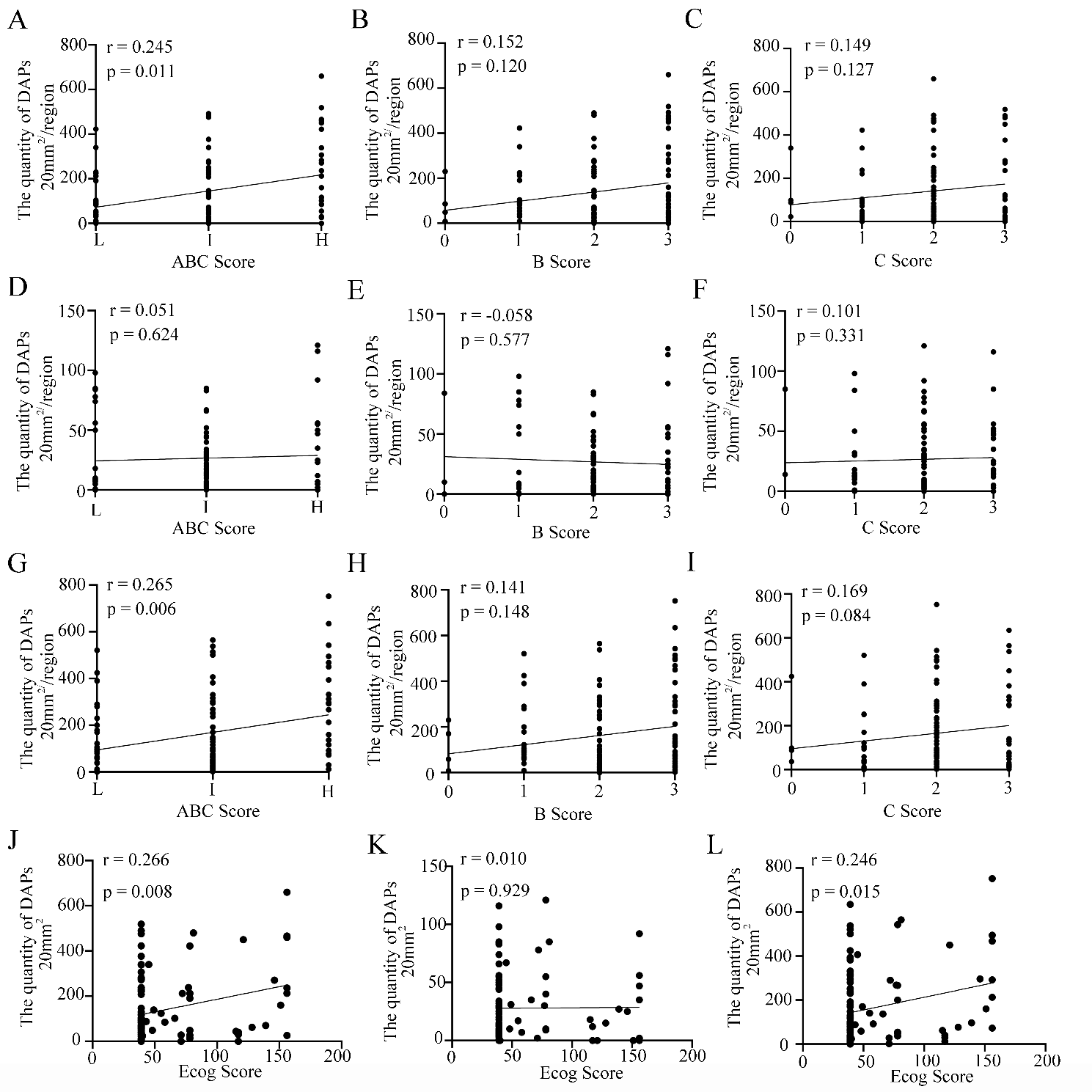


**Figure S2. Correlation between DAPs and ABC scores, ECog scores in human brain. A** The correlation between the quantity of DAPs in cerebral cortex and ABC score. *p* = 0.011, r = 0.245, significant positive correlation, by Spearman correlation. **B** The correlation between the quantity of DAPs in cerebral cortex and B score. *p* > 0.05, no significant correlation, by Spearman correlation. **C** The correlation between the quantity of DAPs in cerebral cortex and the C score. *p* > 0.05, no significant correlation, by Spearman correlation. **D** The correlation between the quantity of DAPs in hippocampus and ABC score. *p* > 0.05, no significant correlation, by Spearman correlation. **E** The correlation between the quantity of DAPs in hippocampus and B score. *p* > 0.05, no significant correlation, by Spearman correlation. **F** The correlation between the quantity of DAPs in hippocampus and the C score. *p* > 0.05, no significant correlation, by Spearman correlation. **G** The correlation between the quantity of DAPs in the whole brain and ABC score. *p* = 0.006, r = 0.265, significant positive correlation, by Spearman correlation. **H** The correlation between the quantity of DAPs in the whole brain and B score. *p* > 0.05, no significant correlation, by Spearman correlation. **I** The correlation between the quantity of DAPs in the whole brain and the C score. *p* > 0.05, no significant correlation, by Spearman correlation. In **A-C** and **G-I**, 106 samples were included. In **D-F**, 95 samples were included. **J** The correlation between the quantity of DAPs in cerebral cortex and ECog score. *p* = 0.008, r = 0.266, significant positive correlation, by Spearman correlation. **K** The correlation between the quantity of DAPs in hippocampus and ECog score. **L** The correlation between the quantity of DAPs in the whole brain and ECog score. *p* = 0.015, r = 0.246, significant positive correlation, by Spearman correlation. In **J** and **L**, 98 samples were included. In **K**, 87 samples were included.


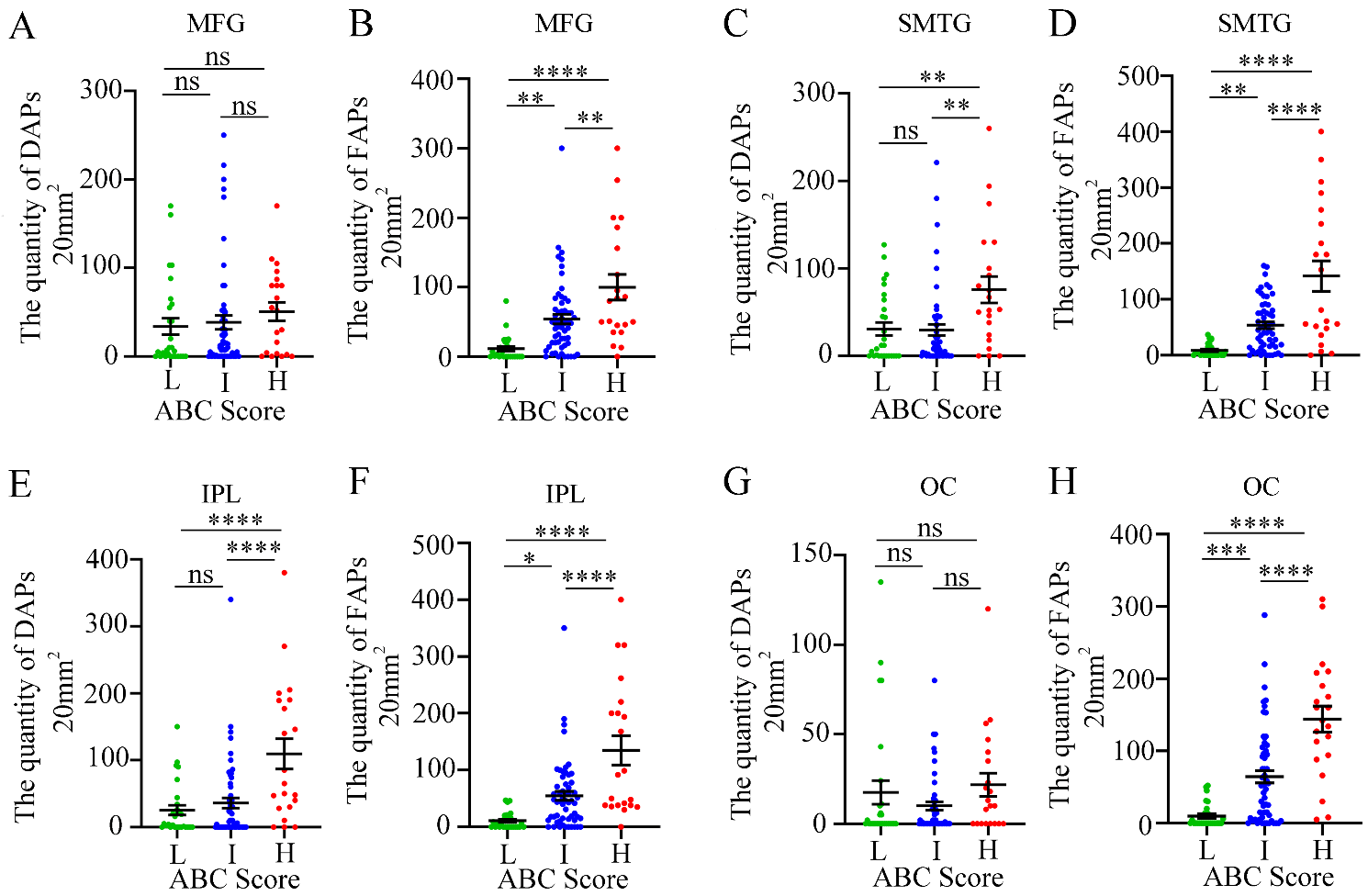


**Figure S3. Distribution of different types of Aβ plaques across cortical functional areas. A** The quantity of DAPs in middle frontal gyrus across different ABC score groups. **B** The quantity of FAPs in middle frontal gyrus across different ABC score groups. **C** The quantity of DAPs in superior and middle temporal gyri across different ABC score groups. **D** The quantity of FAPs in superior and middle temporal gyri across different ABC score groups. **E** The quantity of DAPs in inferior parietal lobule across different ABC score groups. **F** The quantity of FAPs in inferior parietal lobule across different ABC score groups. **G** The quantity of DAPs in occipital cortex across different ABC score groups. **H** The quantity of FAPs in occipital cortex across different ABC score groups. In **A-H**, “L” group (n = 28), “I” group (n = 57) and “H” group (n = 21). MFG: middle frontal gyrus, SMTG: superior and middle temporal gyri, IPL: inferior parietal lobule, OC: occipital cortex. **p* < 0.05, ***p* < 0.01, ****p* < 0.001, *****p* < 0.0001, by one-way ANOVA followed by Scheffe’s post-hoc test.

**
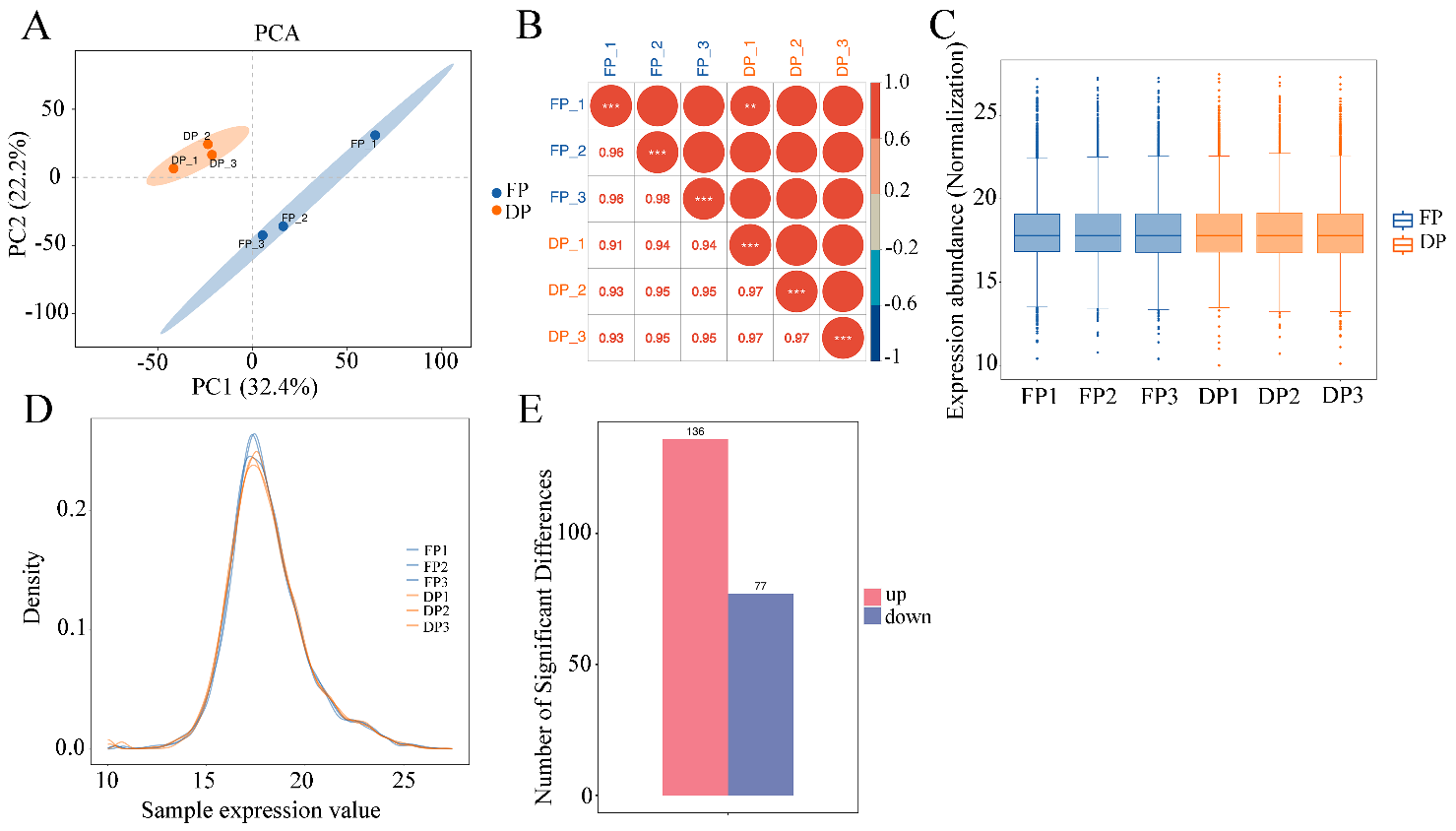
**

**Figure S4. Trusted protein analysis and data quality control in proteomics. A** Principal Component Analysis (PCA) score plot using the expression levels of reliable proteins. The horizontal axis represents the PC1, which is the first principal component explained rate, and the vertical axis represents the PC2, which is the second principal component explained rate. Each point in the graph represents a sample. **B** The graph for sample correlation analysis of reliable proteins. The upper triangle, located to the right of the diagonal, uses red to indicate positive correlation and blue to indicate negative correlation. It visually represents the magnitude of the correlation coefficient for each pair. The lower triangle, situated to the left of the diagonal, displays numerical values for the correlation coefficient and utilizes color coding to differentiate between positive and negative correlations. Additionally, distinct colors are used for sample labels to denote different groupings. **C** The boxplot depicting the expression levels of reliable proteins. The height of the box to some extent reflects the degree of data variability. A flatter box indicates more concentrated data, while shorter whiskers also indicate more concentrated data. The greater the deviation of the median from the center position between the upper and lower quartiles, the stronger the skewness of the distribution. **D** The density plot depicting the expression levels of reliable proteins. Each curve represents the probability distribution of the expression values for that sample. A higher peak indicates a denser data distribution at that point, reflecting higher density. **E** The bar chart of differentially expressed proteins. The selection criteria for differential proteins are as follows: Foldchange ≥ 1.5 or Foldchange ≤ 1/1.5 and *p* < 0.05.

**
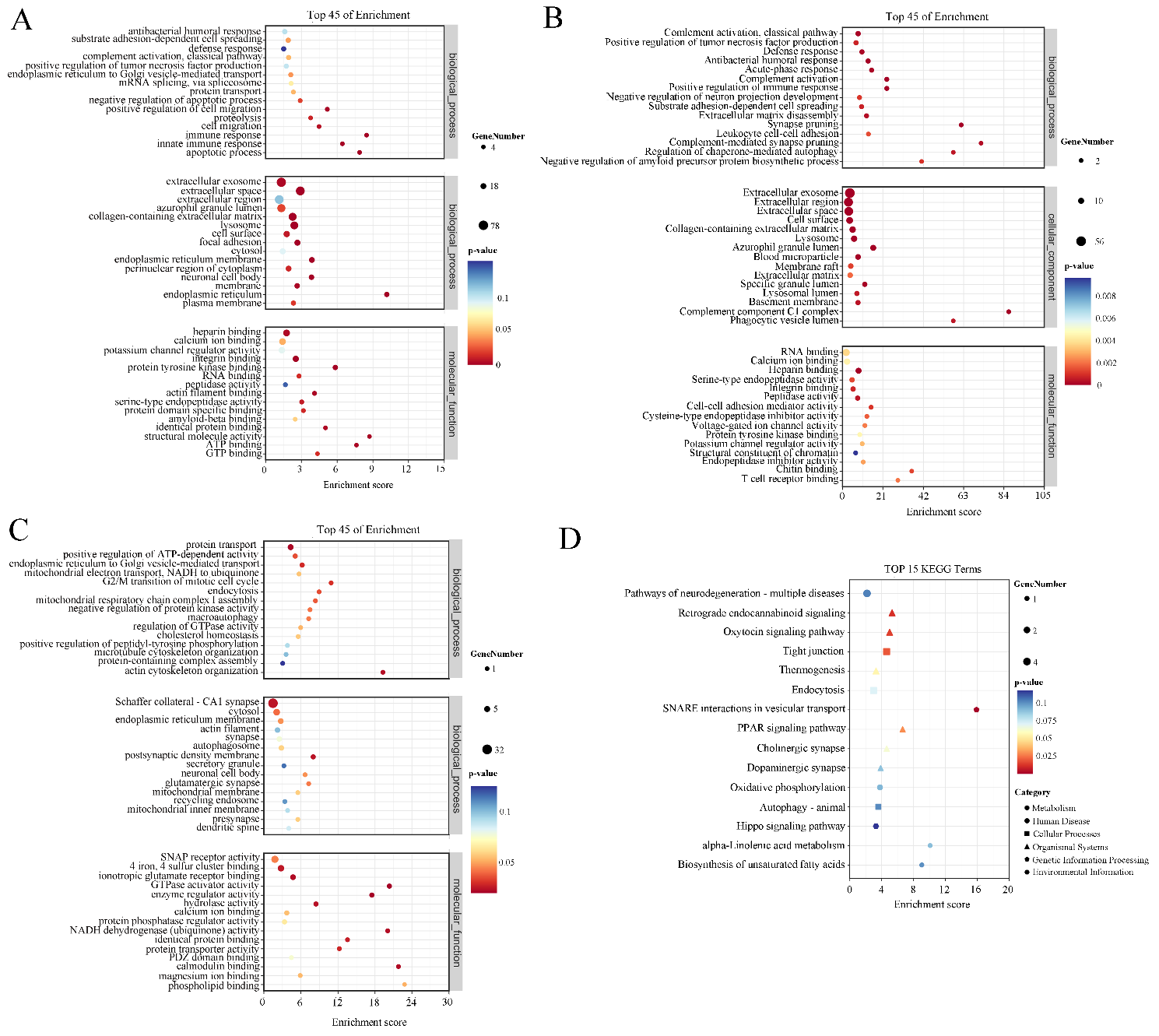
**

**Figure S5. Enrichment analyses of differentially expressed proteins in human brain FAPs versus DAPs. A** The top 15 GOBP, 15 GOCC and 15 GOMF pathways with significantly enriched differentially expressed proteins in FAPs and DAPs. **B** The top 15 GOBP, 15 GOCC and 15 GOMF pathways with significantly enriched upregulated proteins in FAPs. **C** The top 15 GOBP, 15 GOCC and 15 GOMF pathways with significantly enriched downregulated proteins in FAPs. **D** Top 15 enriched KEGG pathways of downregulated proteins in FAPs. In **A-D**, the horizontal axis represents the enrichment score, and the vertical axis represents the top term information. The bigger the bubble, the more proteins it contains, and the color of the bubble changes from blue to red, with the redder color indicating a smaller p-value and a greater significance.

**
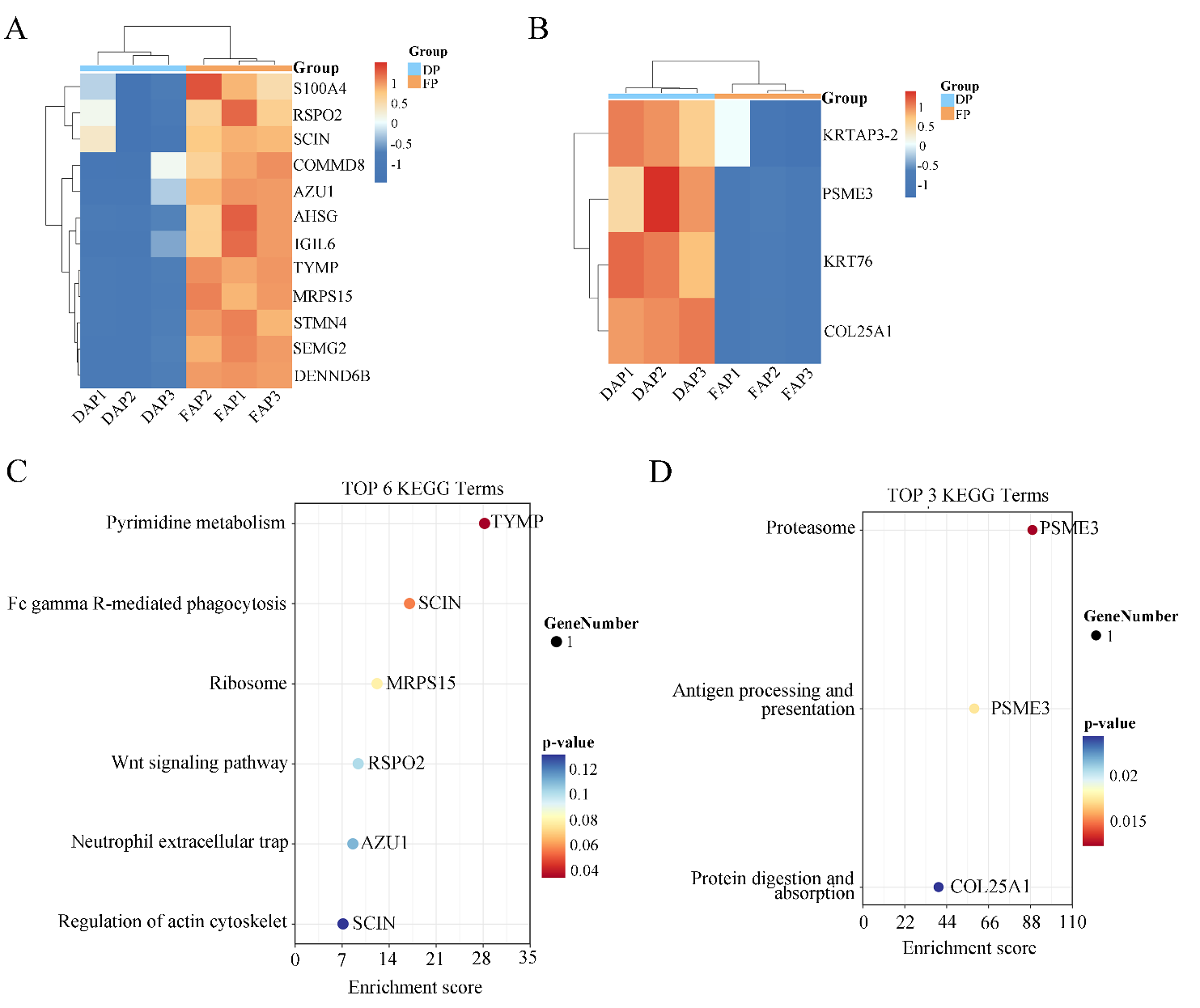
**

**Figure S6. Proteins that are only expressed in FAPs or DAPs. A** Heatmap of proteins exclusively expressed in FAPs. **B** Heatmap of proteins exclusively expressed in DAPs. **C** Top 6 enriched KEGG pathways of FAP-specific proteins (bubble chart). **D** Top 3 enriched KEGG pathways of DAP-specific proteins (bubble chart). In **C-D**, the horizontal axis represents the enrichment score, and the vertical axis represents the top term information. The bigger the bubble, the more proteins it contains, and the color of the bubble changes from blue to red, with the redder color indicating a smaller p-value and a greater significance.

**
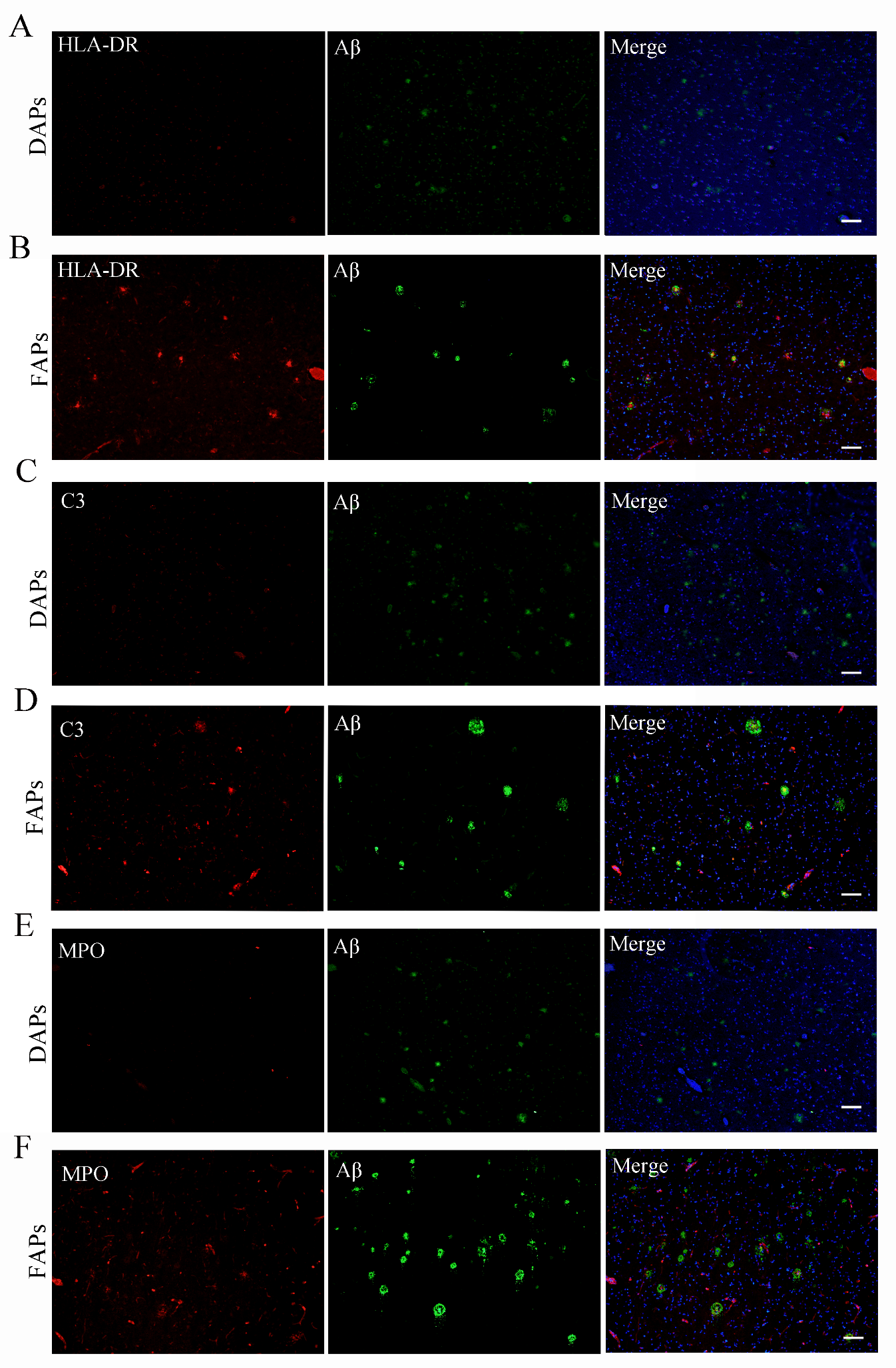
**

**Figure S7. The co-localization of different types of Aβ plaques with characteristic proteins in the human brain cortex under low-magnification microscopy.** **A-B** Representative images of double immunofluorescence staining for HLA-DR and DAPs **(A)** or FAPs **(B)** in human brain. **C-D** Representative images of double immunofluorescence staining for C3 and DAPs **(C)** or FAPs **(D)** in human brain. **E-F** Representative images of double immunofluorescence staining for MPO and DAPs **(E)** or FAPs **(F)** in human brain. In **A**-**F**, scale bar = 100 μm.

**
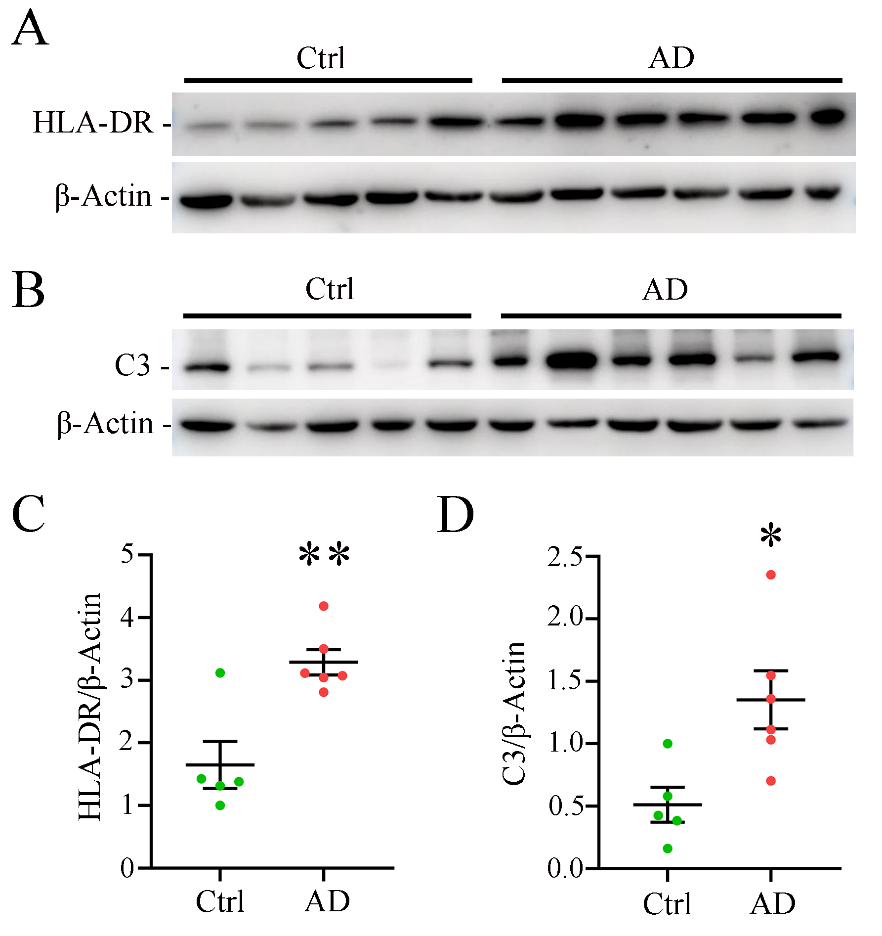
**

**Figure S8. HLA-DR and C3 significantly increased in the AD human brain cortex. A** Western blot of HLA-DR in the human brain cortex. **B** Western blot of C3 in the human brain cortex. **C** The relative expression levels of HLA-DR with β-Actin as the internal reference in the human brain cortex in different groups. **D** The relative expression levels of C3 with β-Actin as the internal reference in the human brain cortex in different groups. In **C** and **D**, data are shown as the mean ± SEM. **p* < 0.05, ***p* < 0.01, by Student's t test. Ctrl group: ABC score “N” and “L” (n = 5). AD group: ABC score “I” and “H” (n = 6).


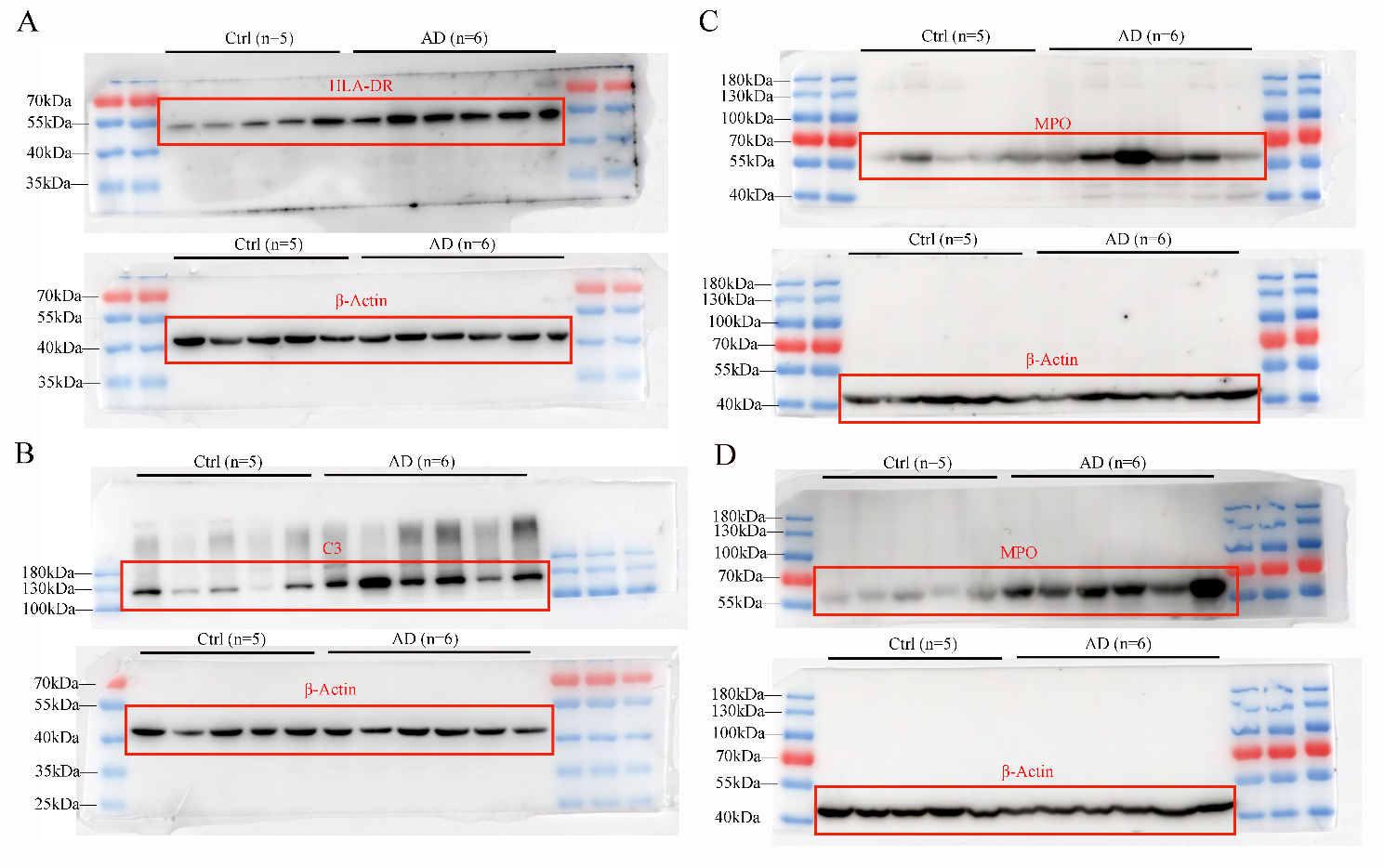


**Figure S9.** **The original western blotting images including molecular weight size markers and sample loading information. A** The original western blotting images of Figure S8A. **B** The original western blotting images of Figure S8B. **C** The original western blotting images of Figure 6A. **D** The original western blotting images of Figure 6D.

**Supplementary Tables**

**Supplementary Table 1. Detailed information on all donors.**

| No | Gender | Ages | PMD（h） | ABC score | A score | B score | C score | ECog score |
| --- | --- | --- | --- | --- | --- | --- | --- | --- |
| Sample01 | F | 87 | 5 | I | 1 | 2 | 2 | null |
| Sample02 | M | 88 | 6.5 | I | 2 | 2 | 2 | null |
| Sample03 | M | 81 | 12 | L | 1 | 1 | 1 | null |
| Sample04 | F | 85 | 4.5 | I | 3 | 2 | 3 | 81 |
| Sample05 | F | 95 | 5 | I | 2 | 3 | 2 | 39 |
| Sample06 | F | 85 | 7 | I | 2 | 2 | 0 | 39 |
| Sample07 | M | 85 | 7.5 | L | 1 | 1 | 2 | 39 |
| Sample08 | M | 79 | 6.5 | H | 3 | 3 | 3 | 39 |
| Sample09 | M | 84 | 5 | I | 2 | 3 | 2 | null |
| Sample10 | M | 90 | 18 | I | 2 | 3 | 3 | null |
| Sample11 | F | 98 | ＞24 | I | 2 | 3 | 2 | null |
| Sample12 | M | 80 | ＞24 | I | 1 | 2 | 2 | 39 |
| Sample13 | M | 92 | 16.5 | I | 2 | 2 | 2 | null |
| Sample14 | F | 86 | 6.3 | I | 3 | 2 | 3 | 55 |
| Sample15 | F | 80 | 18 | I | 2 | 2 | 2 | 71 |
| Sample16 | F | 86 | 4.5 | I | 1 | 3 | 2 | 39 |
| Sample17 | F | 100 | 3 | I | 1 | 2 | 2 | 45 |
| Sample18 | M | 73 | 18 | L | 1 | 0 | 1 | 39 |
| Sample19 | M | 78 | 8 | L | 2 | 0 | 1 | 110 |
| Sample20 | M | 87 | 11 | H | 3 | 3 | 2 | 58 |
| Sample21 | F | 78 | 20 | L | 1 | 0 | 1 | 48 |
| Sample22 | F | 77 | 21 | L | 2 | 1 | 1 | 39 |
| Sample23 | F | 87 | ＞24 | I | 1 | 3 | 2 | 40 |
| Sample24 | M | 90 | null | L | 1 | 1 | 2 | 39 |
| Sample25 | M | 72 | null | H | 3 | 3 | 3 | 121 |
| Sample26 | M | 68 | 1.5 | L | 1 | 1 | 0 | 39 |
| Sample27 | F | 83 | 3 | I | 2 | 3 | 3 | 115 |
| Sample28 | F | 80 | 13 | L | 1 | 1 | 2 | 72 |
| Sample29 | F | 81 | 4 | L | 1 | 1 | 1 | 39 |
| Sample30 | M | 60 | 20 | L | 1 | 1 | 0 | 43 |
| Sample31 | F | 91 | 3 | H | 3 | 3 | 3 | 66 |
| Sample32 | M | 83 | 4.5 | H | 3 | 3 | 2 | 151 |
| Sample33 | M | 91 | 7 | I | 2 | 2 | 2 | 77 |
| Sample34 | M | 90 | 24 | L | 1 | 1 | 2 | 39 |
| Sample35 | F | 89 | 21 | L | 1 | 2 | 1 | 39 |
| Sample36 | F | 70 | 4.5 | L | 1 | 2 | 1 | 39 |
| Sample37 | M | 86 | ＞24 | L | 1 | 1 | 2 | 39 |
| Sample38 | F | 86 | 8 | I | 2 | 2 | 2 | 39 |
| Sample39 | F | 84 | 4 | I | 3 | 2 | 3 | 39 |
| Sample40 | F | 86 | 18 | I | 2 | 2 | 1 | 39 |
| Sample41 | M | 86 | 10 | L | 1 | 2 | 1 | 39 |
| Sample42 | M | 86 | 20.5 | L | 1 | 1 | 2 | 39 |
| Sample43 | F | 91 | 12 | I | 2 | 3 | 2 | 39 |
| Sample44 | M | 91 | 16.5 | I | 3 | 2 | 3 | 128 |
| Sample45 | M | 91 | 18 | I | 1 | 3 | 2 | 39 |
| Sample46 | F | 81 | 8 | H | 3 | 3 | 3 | 156 |
| Sample47 | F | 90 | ＞24 | H | 3 | 3 | 3 | 146 |
| Sample48 | M | 74 | 2.5 | L | 1 | 1 | 2 | 78 |
| Sample49 | M | 81 | 28 | I | 1 | 2 | 2 | 71 |
| Sample50 | M | 92 | ＞24 | I | 1 | 2 | 2 | 39 |
| Sample51 | M | 70 | 18.5 | L | 1 | 0 | 2 | 39 |
| Sample52 | M | 93 | 6 | I | 3 | 2 | 3 | 39 |
| Sample53 | F | 98 | 7.5 | L | 1 | 2 | 1 | 39 |
| Sample54 | M | 81 | 17.6 | L | 1 | 2 | 1 | 39 |
| Sample55 | M | 80 | 6 | L | 2 | 1 | 1 | 39 |
| Sample56 | M | 80 | ＞24 | I | 3 | 2 | 3 | 39 |
| Sample57 | M | 94 | 9.5 | I | 2 | 3 | 2 | 117 |
| Sample58 | M | 78 | 13.5 | H | 3 | 3 | 2 | 156 |
| Sample59 | F | 88 | 7 | I | 2 | 2 | 2 | 139 |
| Sample60 | M | 81 | 16 | I | 2 | 2 | 3 | 39 |
| Sample61 | M | 77 | 4 | I | 2 | 2 | 3 | 39 |
| Sample62 | M | 79 | 8 | I | 1 | 2 | 2 | 39 |
| Sample63 | F | 86 | 5.5 | I | 2 | 3 | 3 | 78 |
| Sample64 | F | 71 | 18 | I | 2 | 2 | 3 | 39 |
| Sample65 | M | 97 | 18 | I | 2 | 2 | 2 | 39 |
| Sample66 | F | 93 | 2 | I | 1 | 2 | 2 | 39 |
| Sample67 | M | 98 | 3 | H | 3 | 3 | 3 | 117 |
| Sample68 | F | 75 | 8 | I | 3 | 2 | 2 | 39 |
| Sample69 | M | 95 | 13 | L | 1 | 2 | 1 | 39 |
| Sample70 | M | 94 | 4.3 | I | 1 | 2 | 2 | 39 |
| Sample71 | F | 93 | 7.3 | I | 2 | 2 | 2 | 39 |
| Sample72 | M | 88 | 3.3 | H | 3 | 3 | 2 | 39 |
| Sample73 | M | 80 | 22.5 | I | 2 | 2 | 1 | 39 |
| Sample74 | F | 88 | 4.5 | I | 1 | 3 | 2 | null |
| Sample75 | M | 87 | ＞24 | I | 3 | 2 | 3 | 39 |
| Sample76 | F | 85 | 21 | I | 3 | 2 | 3 | 39 |
| Sample77 | F | 72 | 2.5 | L | 1 | 1 | 2 | 39 |
| Sample78 | M | 79 | 3.5 | L | 2 | 1 | 2 | 39 |
| Sample79 | M | 90 | 5.5 | I | 2 | 2 | 2 | 39 |
| Sample80 | M | 85 | 23 | H | 3 | 3 | 3 | 39 |
| Sample81 | M | 73 | 3 | L | 2 | 1 | 2 | 39 |
| Sample82 | M | 94 | 5.5 | I | 2 | 2 | 1 | 39 |
| Sample83 | M | 78 | 5.5 | L | 2 | 1 | 2 | 39 |
| Sample84 | F | 88 | 20 | I | 1 | 2 | 2 | 39 |
| Sample85 | F | 80 | 8 | I | 3 | 2 | 2 | 49 |
| Sample86 | M | 63 | 3 | I | 1 | 2 | 2 | 39 |
| Sample87 | F | 80 | ＞24 | I | 2 | 2 | 2 | 39 |
| Sample88 | F | 87 | 5 | I | 2 | 2 | 2 | 39 |
| Sample89 | F | 90 | 3.5 | H | 3 | 3 | 3 | 39 |
| Sample90 | F | 71 | 9.5 | I | 3 | 2 | 2 | 39 |
| Sample91 | M | 89 | 4.5 | H | 3 | 3 | 3 | 39 |
| Sample92 | F | 92 | 5.5 | I | 2 | 2 | 1 | 39 |
| Sample93 | M | 89 | 3 | H | 3 | 3 | 2 | 39 |
| Sample94 | M | 85 | 26 | H | 3 | 3 | 3 | 156 |
| Sample95 | M | 89 | 4.5 | H | 3 | 3 | 2 | 156 |
| Sample96 | M | 86 | ＞24 | I | 1 | 2 | 2 | 39 |
| Sample97 | M | 93 | null | H | 3 | 3 | 2 | 117 |
| Sample98 | F | 58 | 10 | L | 1 | 1 | 0 | 39 |
| Sample99 | F | 88 | 12 | H | 3 | 3 | 2 | 78 |
| Sample100 | M | 88 | 4.5 | I | 1 | 2 | 2 | 78 |
| Sample101 | F | 95 | 7 | H | 3 | 3 | 2 | 78 |
| Sample102 | M | 71 | 10 | L | 1 | 1 | 1 | 39 |
| Sample103 | F | 85 | 6 | I | 2 | 2 | 2 | 78 |
| Sample104 | F | 89 | 4.5 | H | 3 | 3 | 2 | 156 |
| Sample105 | F | 94 | 7 | H | 3 | 3 | 2 | 156 |
| Sample106 | M | 93 | 23 | I | 3 | 2 | 1 | 39 |
